# Exploring the property space of periodic cellular structures based on crystal networks

**DOI:** 10.1073/pnas.2003504118

**Published:** 2021-02-08

**Authors:** Thomas S. Lumpe, Tino Stankovic

**Affiliations:** ^a^Department of Mechanical and Process Engineering, Engineering Design and Computing Laboratory, ETH Zurich, 8092 Zurich, Switzerland

**Keywords:** cellular structures, crystal networks, extremal materials, numerical homogenization

## Abstract

The properties of periodic cellular structures strongly depend on the regular spatial arrangement of their constituent base materials and can be controlled by changing the topology and geometry of the repeating unit cell. Recent advances in three-dimensional (3D) fabrication technologies more and more expand the limits of fabricable real-world architected materials and strengthen the need of novel microstructural topologies for applications across all length scales and fields in both fundamental science and engineering practice. Here, we systematically explore, interpret, and analyze publicly available crystallographic network topologies from a structural point of view and provide a ready-to-use unit cell catalog with more than 17,000 unique entries in total. We show that molecular crystal networks with atoms connected by chemical bonds can be interpreted as cellular structures with nodes connected by mechanical bars. By this, we identify new structures with extremal properties as well as known structures such as the octet-truss or the Kelvin cell and show how crystallographic symmetries are related to the mechanical properties of the structures. Our work provides inspiration for the discovery of novel cellular structures and paves the way for computational methods to explore and design microstructures with unprecedented properties, bridging the gap between microscopic crystal chemistry and macroscopic structural engineering.

## Significance

Finding genuine novelty in cellular structures is inherently difficult due to the numerous possible topological and geometrical configurations and their complex mechanical and physical interrelations. Here, we draw inspiration from the incredibly rich collection of crystallographic periodic networks that we interpret from a structural point of view to identify and design novel cellular structures with unique properties. We provide a ready-to-use catalog with more than 17,000 unique entries and show how crystallographic symmetries relate to their mechanical properties. Our work provides a foundation to support future applications in science and engineering, ranging from mechanical and optical metamaterials, over bone tissue engineering, to the design of electrochemical devices.

Crystallographers were among the first to study the organizing principles behind the formation of patterns and distinct network topologies in solid materials and to relate them to their overall macroscopic properties (1, 2). Similar to these crystallographic networks, the properties of periodic cellular structures can be designed by controlling the architecture of the repeating unit cell (3). This results in the purposeful design of cellular structures with advanced macroscopic mechanical properties such as negative Poisson’s ratios (4, 5), excellent strength-to-weight ratios (6, 7), and controlled instabilities (8, 9). While finding novel microstructures has proven essential for the development of future applications and technologies (10, 11), many architected cellular structures in engineering (12, 13), fundamental science (14–16), and advanced manufacturing (17, 18) are based on a small selection of well-studied designs. Finding genuine novelty in cellular structures is inherently difficult due to the numerous possible topological and geometrical configurations and their complex mechanical and physical interrelations. To address these difficulties, recent computational database-driven methods (19, 20) support the design of cellular structures by generating parametrized unit cells from initial datasets of different topologies. These initial topologies are often still based on well-known structures from literature, but are further modified by computational methods such as search and interpolation algorithms or topology optimization to populate the design space more extensively (19, 20). However, the discovery of novel structures necessitates that the formalization of the underlying search space possesses this intrinsic novelty as well. Pure topology optimization approaches, which can provide this novelty, are often computationally very expensive, especially in the three-dimensional (3D) case, and exhibit nonuniqueness and a strong starting-point sensitivity of the solutions (21). This makes the results highly sensitive toward external constraints, where alternative solutions with similar properties but different topologies could drastically improve the overall quality of the results. Hence, formalizing prior knowledge and variety to control and increase the range of viable solutions or to find alternative configurations could be highly beneficial for both database-driven methods and topology optimization approaches.

**Fig. 1.**
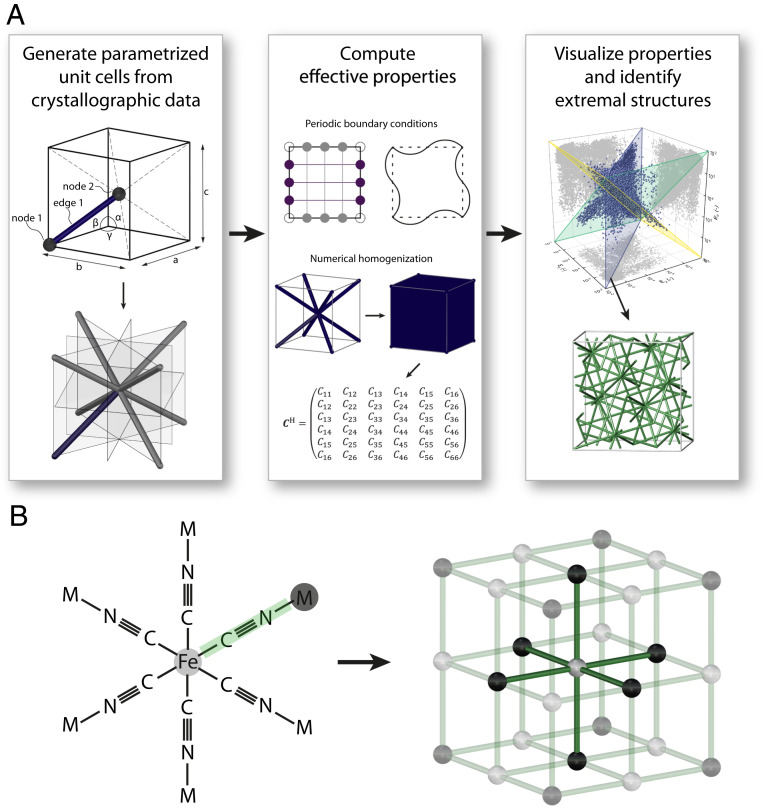
(*A*) Overview of the method. In the first step, we obtain the crystallographic data of 17,087 unique crystal networks from the two publicly available databases RCSR and EPINET. We use the program SYSTRE (32) to obtain the parametrized unit cells with explicit nodal positions and bar connectivities. Next, a numerical homogenization approach with periodic boundary conditions is used to compute the effective properties of all unit cells at the same relative density. Finally, we visualize the property range of all structures and identify structures with interesting or extremal properties. (*B*) The chemical description of a minimum unit of bimetallic Prussian Blue-like α-polonium (*Left*) and its corresponding 3D network (*Right*), where Fe and M atoms are interpreted as nodes and C–N bonds are interpreted as bars connecting the nodes. Reprinted from ref. 31. Copyright (2005), with permission from Elsevier.

To facilitate the design of novel microstructural architectures and to complement existing computational methods, in this work we systematically explore, interpret, and analyze the incredibly rich collection of crystallographic periodic network topologies from a structural point of view. We provide a ready-to-use unit cell catalog with 17,087 unique entries in total, offering a source of knowledge and inspiration for engineers and scientists by substantially extending the limited set of well-known unit cells from literature with crystal-network-based topologies. Our catalog and the results in this paper are based on the two publicly accessible databases Reticular Chemistry Structure Resource (RCSR) (22, 23) and Euclidean Patterns in Non-Euclidean Tilings (EPINET) (24, 25). Due to the generality of our approach, it is readily applicable to the analysis of other crystallographic databases or the generation of completely new structures based on the underlying mathematical principles. To generate our catalog, we apply an integrated design and simulation methodology based on numerical homogenization, summarized in Fig. 1*A*. We explore the resulting mechanical property space and show how common geometrical features relate to the structures at the extremes of the mapped spaces. We further identify both novel structures with extremal properties and well-known structures from literature such as the octet-truss or the Kelvin cell and show how crystallographic symmetries relate to their mechanical properties. To make our work directly accessible for future applications, we provide the full text catalog along with visualizations of all structures and 360° surface plots of the Young’s modulus, respectively (26, 27). Moreover, our catalog includes a variety of structures that form the basis for fundamental, interdisciplinary research, including the design of scaffolds for bone tissue regeneration (28), microstructure-based high-capacity lithium-ion batteries (29), or bioinspired robust lattices (30), as well as a large selection of novel structures with equivalent mechanical properties, which can aid further developments or spark innovation across many disciplines in both science and engineering.

## Crystal Networks as Cellular Structures

In crystal chemistry, crystalline solids or crystals are solid materials with regular periodic microstructures. Their spatial arrangement is governed by the atomic forces between atoms, resulting in the formation of molecular networks. In theory, these molecular networks extend infinitely, and the representation of the intrinsic organizing principle of this microstructure is called the associated crystal network, or simply net (31). Due to the periodicity of crystal networks, their spatial realization can be fully described by the smallest possible set of constituent elements, the unit cell. Since this generative principle of a minimum repeatable unit cell is present in both crystallographic structures and architected cellular structures, we can establish an analogy between the two fields. Hence, following the crystallographic network representation of atoms connected by different bonds, a crystal network can likewise be interpreted in a structural engineering context as a space-filling macroscopic cellular structure with nodes connected by solid bars (3). This analogy is schematically shown in Fig. 1*B*, which relates the bimetallic Prussian Blue-like α-polonium (left) and its corresponding cubic net (right) (adapted from ref. 31).

The periodicity of an infinite crystal network allows for a compact encoding of its structural features based on the overall geometry of the unit cell and the position of the nodes and edges in the unit cell. The geometry of a 3D unit cell is specified by one of the 14 translational symmetry preserving Bravais lattices, which are spanned by three noncollinear base vectors describing the shape and the size of the unit cell. The spatial realization of a crystal network in the Euclidean space, i.e., the position of nodes and the edges connecting the nodes, is directly linked to the nodal positions of the atoms in the unit cell and the chemical bonds between them, and usually reflects the embedding with the highest symmetry possible. In the simplest case, the embedding is obtained by joining the nearest-neighbor nodes to form the edges, which corresponds to a sphere packing with ideally equal edge lengths. For more complex structures, the maximum symmetry embedding might be of lower symmetry, and equal edge lengths are not always possible. The sets of symmetry operations that constitute the final realizations of the nets are summarized in 230 space groups in 3D and are categorized with respect to point-symmetry operations and the underlying crystallographic systems. Since all possible configurations of periodic structures in the Euclidean space conform to one of the space groups, crystallography provides us with a comprehensive and concise mathematical description that we can use to construct and design cellular structures. In the this work, we use the program SYSTRE (32) with the relaxed barycentric placement method (33) to compute the realizations of the structures in our catalog.

The two publicly accessible databases used in this work, the RCSR database (22) and the EPINET database (23), centralize crystallographic data of numerous materials and compounds and further provide information about the structure of the underlying nets. We evaluate in total 17,087 unique nets that represent the spatial arrangement of molecules found in real materials and compounds, but also hypothetical nets that have not been experimentally observed yet. To establish a link between the names of the structures in our work and their respective topological features, we provide a descriptive name for each structure in the form of cub_Z12.0_E19, which includes the crystal system (triclinic, monoclinic, orthorhombic, tetragonal, trigonal, hexagonal, or cubic) specified by the first three letters, the average connectivity $Z_{\text{a}}$ as an important parameter to categorize the topology of a structure, and a unique identifier that consists of the letter E or R and an ordinal number, which links the structures to the two original databases and facilitates traceability.

## The Mechanical Property Space of Cellular Structures in the Catalog

We compute the linear-elastic effective material properties of all structures in the catalog using a numerical homogenization approach (34). In 3D, six independent load cases are required to determine the homogenized effective stiffness matrix $C^{\text{H}}$. To compare the mechanical properties of different structures, the dimensions of a unit cell are uniformly scaled at a constant bar radius r=0.1 mm such that a relative density of $\bar{\rho }=0.01$ is obtained. A solid base material with the Young’s modulus $E_{\text{s}}=1\;\text{MPa}$ and the Poisson’s ratio $\nu_{\text{s}}=0.3$ is assumed. We verify the homogenization framework by comparison to analytical results from literature and experimental results from uniaxial compression tests on selected structures (*SI Appendix*, Fig. S1). To estimate the property range of the cellular structures in the catalog and to identify topologies with extreme mechanical behavior, we compute and visualize the homogenized effective Young’s moduli, shear moduli, and Poisson’s ratios of all structures. The text-based unit cell catalog (26) includes geometrical and mechanical information of all structures. The additional dataset in ref. 25 further includes images of all structures and the respective 360° elastic surfaces of the orientation-dependent Young’s modulus. More details can be found in *Methods* and in *SI Appendix*.

**Fig. 2.**
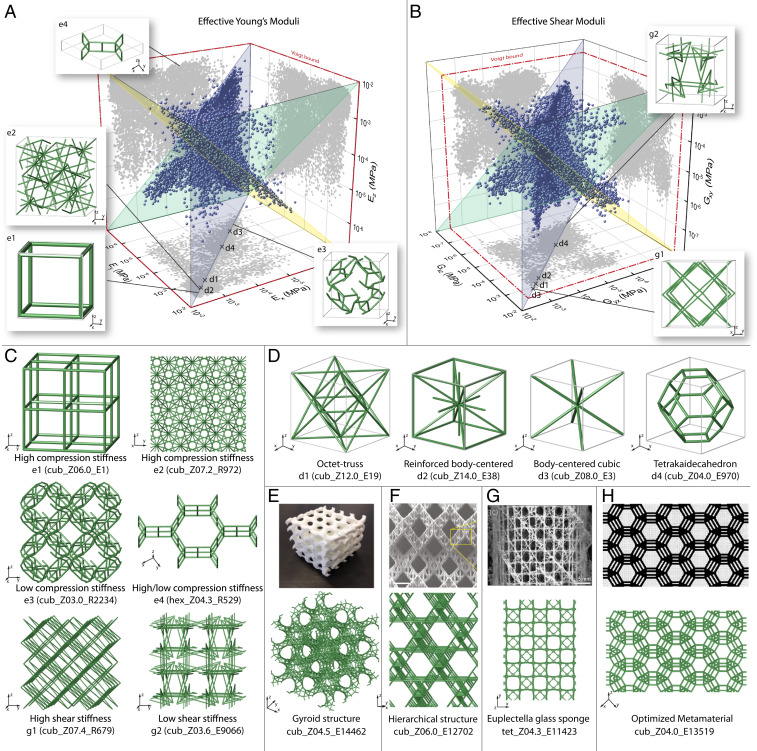
Effective mechanical properties of all 17,087 structures in the catalog and selected examples. (*A*) Effective Young’s modulus in the three main directions of the global *x*, *y*, *z* coordinate system and the values projected on the *yz* plane, the *xz* plane, and the *xy* plane, respectively. The insets e1–e4 show unit cells of structures that contain geometrical and topological patterns representative of the respective region in the plot. For simplicity, the positions are only depicted in one of the projection planes. The three colored transparent planes indicate regions with the same properties in two directions. (*B*) Effective shear modulus in the three main directions of the global coordinate system and the values projected on the *yz* plane, the *xz* plane, and the *xy* plane, respectively. (*C*) Axonometric view of 2 × 2 × 2 unit cells of the structures e1–e4 and g1–g2. (*D*) Unit cells of well-known structures from literature that are also found in the catalog. Their mechanical properties are indicated by the cross-markers in *A* and *C*. For simplicity, the markers are only shown in the *xy* projection plane. (*E*–*H*) Examples of complex structure from literature, where similar structures are found in the catalog. (*E*) Reproduced from ref. 43, which is licensed under CC BY 4.0. (*F*) Reproduced by permission from ref. 46. Springer Nature: Nature Materials, Copyright 2016. (*G*) From ref. 47. Reprinted with permission from AAAS. (*H*) Reprinted from ref. 21. Copyright (2000), with permission from Elsevier.

Fig. 2 *A* and *B* show the effective Young’s modulus E and the effective shear modulus G on a logarithmic scale in the three main directions of the global Cartesian coordinate system *x*, *y*, *z*, and the properties projected onto the *xy* plane, the *yz* plane, and the *xz* plane, respectively. The range of properties in the three directions spans several orders of magnitude between $10^{-8}$ and $10^{-3}$. We use the three transparent bisecting planes to indicate regions of equal properties in two of the three main directions. Hence, the intersection of these three planes is a line that contains structures with the same Young’s modulus in all three directions, which is characteristic for cubic and isotropic material behavior. Most structures that we analyze are found on or close to the three bisecting planes and their intersection line, implying that at least two of the three directions are directly linked by structural symmetries. The red dashed–dotted lines indicate the Voigt bounds, which represent the theoretical maximum Young’s modulus and shear modulus. As a function of the base materials’ elastic moduli and the relative densities ($E_{\text{Voigt}}=E_{\text{s}}*\bar{\rho }$ and $G_{\text{Voigt}}=G_{\text{s}}*\bar{\rho }$), they represent the most general bounds for cellular structures (35). A more detailed discussion on the theoretical bounds can be found in *SI Appendix*.

The insets e1 to e4 in Fig. 2*A* and the insets g1 and g2 in Fig. 2*B* show the unit cells of representative structures located near the boundaries of the property space. Fig. 2*C* shows the same structures in a tessellation of 2 × 2 × 2 unit cells. The structures shown in this work do not necessarily represent the structures with the most extreme values found in the catalog but structures in the same regions of interest with similar properties that are better suited to outline the underlying topological and geometrical patterns. For simplicity, the locations of the data points of the structures discussed in Fig. 2 *A* and *B* are indicated only on one of the three projection planes, and the exact values are provided in *SI Appendix*.

The insets e1 and e2 show examples of structures with two frequently found patterns that result in large Young’s moduli in the three main coordinate directions *x*, *y*, *z*. The structure cub_Z06.0_E1 in e1 consists of straight bars along the three directions, which is intuitively expected as the simplest pattern for high unidirectional stiffness. The structure cub_Z07.2_R972 in e2 also has a relatively high stiffness but is significantly more complex with a higher average connectivity at the nodes of $Z_{\text{a}}=7.2$. The average connectivity describes the average number of bars connected at a node in a structure and can be directly related to the mechanical properties. In general, a connectivity of $Z_{\text{a}}\geq 6$ is a necessary, but not sufficient condition for the rigidity of periodic beam structures (36). Rigid structures are associated with stretch-dominated behavior, whereas nonrigid structures show bending-dominated behavior. However, the sufficiency condition for rigidity in ref. 34 is only applicable to the special class of similarly situated structures. Hence, to assess the rigidity of all structures in the catalog and to make them comparable, we derive scaling relationships between the axial stiffness in the main coordinate directions and the relative density of the form $E/E_{\text{s}}∼{\bar{\rho }}^{n}$. The scaling exponent n indicates stretch- (n=1) or bending-dominated (n=2) behavior, respectively (37, 38). A detailed discussion about the rigidity of frameworks and the density scaling is included in *SI Appendix*. For the structure cub_Z07.2_R972, the scaling exponent is n=1.0 and confirms the rigid behavior in the main coordinate directions.

At the lower end of the material property spectrum, the structure cub_Z03.0_R2234 in e3 is representative of structures that are extremely compliant in all three directions, where a low average connectivity of $Z_{\text{a}}<6$ and n=2.0 suggest bending-dominated behavior. Further, the inset e4 shows a structure with strong anisotropic behavior (hex_Z04.3_R529), which is located close to the boundary of the projected property space. The combination of compliant elements such as honeycomb cells (as seen in the tessellation in Fig. 2*C*) and stiff elements such as straight bars enables the decoupling of the elastic properties in different spatial directions and is often found in the boundary regions of the property space. This stiffness anisotropy is also visible in the 360° plots of the elastic surface of the Young’s modulus, which are provided for all structures in ref. 25. We observe that the directions of the maximum stiffness of the structures do not always coincide with the *x*, *y*, *z* directions of the coordinate system, which is for example the case for structures with diagonal bars oriented at 45° with respect to the main axes. These patterns are often found among structures with high shear stiffness such as the structure cub_Z07.4_R679 in inset g1 in Fig. 2*B*, as the diagonal bars directly stiffen the structures in the main shear directions. In contrast to that, structures with low shear stiffness are often characterized by many intertwined members with different orientations and a low average connectivity, which locally produce high bending loads. The inset g2 shows a structure representative for architectures in this region (cub_Z03.6_E9066). A detailed discussion about the stiffness anisotropy of the structures can be found in *SI Appendix*.

## Related Structures from Literature

Some of the examples from the catalog show structures whose features are well-known in the field of cellular materials (12, 39–42). Fig. 2*D* shows four examples of such structures. The mechanical properties of stretch-dominated structures such as the octet-truss (inset d1, cub_Z12.0_E19) and the reinforced body-centered cubic cell (d2, cub_Z14.0_E38) are investigated in many studies and are used in the design of stiff and light structures (12, 39). Likewise, bending-dominated structures with lower stiffness such as the body-centered cubic cell (d3, cub_Z08.0_E3) and the Tetrakaidecahedron (d4, cub_Z04.0_E970), also known as the Kelvin cell, can be found in the catalog (40, 41). The cross-markers in Fig. 2 *A* and *C* indicate the effective Young’s and shear moduli of these four structures, which are the same in the three main directions due to their cubic symmetry.

Fig. 2 *E*–*G* show examples of more complex structures from literature and the related structures found in our catalog, respectively. The first structure in Fig. 2*E* is a triply periodic minimal surface or gyroid structure, which is among others described in ref. 43. The gyroid structure has a high surface-to-volume ratio, a high specific strength, and a high pore connectivity, which makes it especially suitable for biomedical applications. This geometry is also found in an idealized 3D graphene bulk structure (44). The bottom image in Fig. 2*E* shows 2 × 2 × 2 unit cells of the structure cub_Z04.5_E14462. The structure resembles the gyroid structure shown in the upper image, but the solid surfaces are discretized with four-connected and five-connected bars. Since the structures in the EPINET database are based on periodic surfaces, triply periodic minimal surfaces are included as a subclass and are frequently found in the catalog. The discretized structure from the catalog can be interpreted as a hierarchical realization of the initially solid-shell structure, with the bars and the gyroid as the two levels of hierarchy with different characteristic length scales. Hierarchical structures often occur in nature, for example in bone structures, and sparked considerable interest in structural engineering research due to their enhanced mechanical properties (45). Fig. 2*F* (*Top*) shows an example from ref. 46, where a bending-dominated structure at the higher hierarchy level is discretized by smaller bars, forming stretch-dominated structures at the lower hierarchy level. The bottom image of Fig. 2*F* depicts the structure cub_Z06.0_E12702, which resembles the structure from ref. 46 and is defined by a bending-dominated diamond unit cell at the higher hierarchy level. At the lower hierarchy level, each part of the diamond cell is discretized by smaller bars with a connectivity of six and stretch-dominated behavior. A hierarchical system found in nature is shown at the top of Fig. 2*G* as the skeletal system of *Euplectella sp.* The *Euplectella sp.* structure is composed of horizontal, vertical, and diagonal struts arranged in a square checkerboard pattern at the millimeter scale (47). The structure tet_Z04.3_E11423 from the catalog viewed in the *z* direction strongly resembles this pattern and shows that the crystallography-based encoding of the structures not only describes the topology of microscopic molecules but is also able to capture evolutionary patterns found in nature at much bigger length scales. A recent work by ref. 30 also points out that a structure based on *Euplectella sp* and similar to tet_Z04.3_E11423 can be exploited for the design of mechanically robust structures. Fig. 2*H* (*Top*) shows a different type of hierarchical structure whose two-dimensional (2D) realization is the result of an inverse homogenization problem proposed in ref. 21. The structure has a large bulk modulus with a low shear modulus and combines a hexagonal microstructure at the higher hierarchy level with parallel laminatelike bars at the lower hierarchy level. A 3D realization of this distinct pattern is found in our catalog in the structure cub_Z04.0_E13519. This structure is originally a hierarchical gyroid like the example in Fig. 2*E*. However, the laminate pattern becomes visible when the structure is viewed in an isometric projection that aligns with the 45° direction of the shear plane. Although the laminate pattern in this example is only a 2D projection of the multiple layers of the 3D gyroid structure, it nonetheless originates from the application of crystallographic symmetries and tiling and shows that the crystallographic organizing principles can be used to complement the existing computational approaches to optimize the properties of microstructures.

## Poisson’s Ratio

**Fig. 3.**
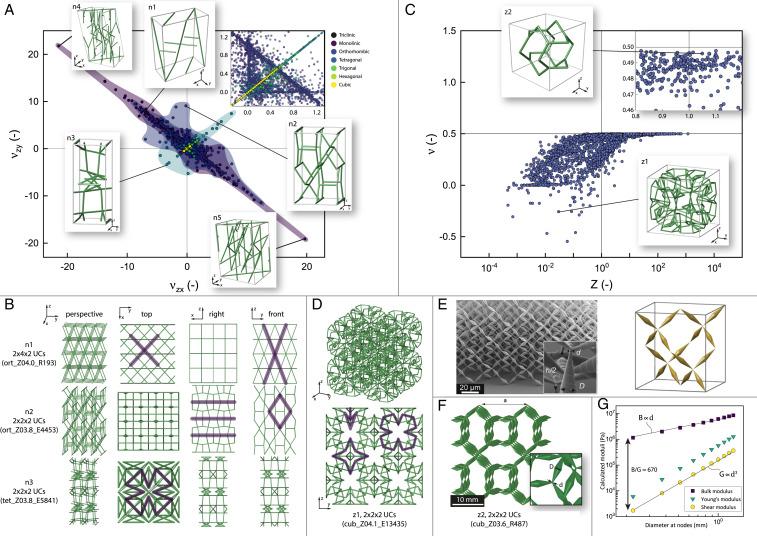
(*A*) Poisson’s ratios $\nu_{zx},\nu_{zy}$ of all 17,087 structures in the catalog and a detailed view of the range −0.2≤ν≤1.3. The highlighted regions visualize the range of properties. The colors of the dots and the regions indicate the different crystal systems. The insets n1–n3 show unit cells of structures that contain geometrical and topological patterns representative of the respective region. The insets n4 and n5 show the structures mon_Z04.0_E8038 and mon_Z04.3_E4603 with the maximum and minimum Poisson’s ratios. (*B*) Different views of multiple unit cells of the structures in the insets n1–n3. The dark lines highlight patterns that are related to the respective mechanical behavior of the structures. (*C*) Poisson’s ratio and Zener anisotropy value of all 641 structures with cubic symmetry. A *Z* value close to 1 indicates isotropic behavior. The inset z1 shows an auxetic structure with cubic symmetry. The inset z2 shows a structure with a Zener value close to 1 and a Poisson’s ratio close to 0.5, which is identified as a possible extremal pentamode material. (*D*) Axonometric view and front view of 2 × 2 × 2 unit cells of the auxetic structure cub_Z04.1_E13435. Dark lines highlight different well-known patterns that cause auxetic behavior. (*E*) Image of the first manufactured pentamode metamaterial and its unit cell based on the diamond structure. The biconical cross-sections of the bars with very small diameters close to the nodes drastically increase the bulk-to-shear modulus ratio. Reprinted from ref. 14, with permission of AIP Publishing. (*F*) An image of 2 × 2 × 2 unit cells of the structure cub_Z03.6_R487 with biconical bars. For comparison, the dimensions of a,d,D are chosen according to ref. 55. (*G*) Effective Young’s modulus, shear modulus, and bulk modulus of the structure cub_Z03.6_R487 for different values of d. The solid lines indicate scaling relations of B∝d and $G\propto d^{3}$ (no fits). The maximum value of B/G≈670 indicates pentamode behavior.

Fig. 3*A* shows the Poisson’s ratios $\nu_{zx}$ and $\nu_{zy}$ of all structures in the catalog (other directions are provided in *SI Appendix*). The inset at the top right in Fig. 3*A* shows the range between −0.2≤ν≤1.3, in which about 91% of all structures in the catalog are located. Furthermore, many structures lie on or close to the two diagonal lines $\nu_{zy}=-\nu_{zx}$ or $\nu_{zy}=\nu_{zx}$, or on the lines $\nu_{zx}=0$ and $\nu_{zy}=0$. To highlight the influence of the space-group-related symmetry, the structures are clustered according to their crystal system. On the microscopic material level, Neumann’s principle states that the symmetry of physical properties is directly linked to the point-group symmetry of the respective crystal (48). Hence, the crystal symmetry reduces the number of independent material constants according to the crystal system. While triclinic materials are fully anisotropic with 21 independent constants, this number is reduced to 3 for cubic materials. The cubic symmetry has the highest possible symmetry and includes the case of isotropic materials with two independent constants, where two of the three constants are coupled. In general, anisotropic materials have no bounds on the Poisson’s ratio (49), which is why the most extreme values are found in structures with the lowest symmetries. This finding is consistent with literature, where extreme elastic mechanical properties are associated with strong material anisotropy (50). Conversely, isotropic structures that represent structures with the highest symmetry are bounded by −1≤ν≤0.5 as imposed by the thermodynamic stability criterion, i.e., the positive definiteness of the stiffness tensor. These limits are valid for all structures with at least cubic symmetry and only one independent value of the Poisson’s ratio $\nu =\nu_{zy}=\nu_{zx}$ (yellow dots in Fig. 3*A*). Since the catalog contains in total only three structures with triclinic symmetry with Poisson’s ratios smaller than 1, their individual markers are not visible in Fig. 3*A*.

Fig. 3*A* (*Insets*) show examples of structures with extreme deformation behavior under a compressive deformation in the *z* direction. The structure ort_Z04.0_R193 in the inset n1 expands in the *y* direction $\left(\nu_{zy}=6.99\right)$ and contracts in the *x* direction $\left(\nu_{zx}=-6.01\right)$. This behavior is enabled by slanted vertical bars that are coupled to slanted horizontal bars. This creates scissorlike structures, as highlighted in the top view and front view in Fig. 3*B*. These coupled slanted columns occur in most of the monoclinic structures with extreme values along the line $\nu_{zy}=-\nu_{zx}$ and are also found in the structures with the most extreme properties, mon_Z04.0_E8038 and mon_Z04.3_E4603, which are shown in the insets n4 and n5. The structure ort_Z03.8_E4453 in inset n2 expands in the *y* direction $\left(\nu_{zy}=9.09\right)$ with a relatively small contraction in the *x* direction $\left(\nu_{zx}=-0.16\right)$. Fig. 3*B* shows that rhombic arrangements of bars account for the expansion in the *y* direction in this structure, while horizontal straight bars prevent large deformations in the *x* direction. Like the previously discussed decoupled stiffness, the combination of stretch-dominated elements such as straight bars and bending-dominated elements such as rhombic or scissorlike arrangements enables the decoupling of compliant deformation modes in anisotropic structures. The inset n3 shows the structure tet_Z03.8_E5841, which contracts in the *x* direction and in the *y* direction simultaneously. This structure is partly auxetic with Poisson’s ratios of $\nu_{zx}=\nu_{zy}=-3.33$. The main part of the unit cell of the structure consists of two coiled chains in the shape of a double helix. The top view of the structure n3 with 2 × 2 × 2 unit cells in Fig. 3*B* reveals the well-known reentrant pattern that causes the auxetic behavior (51). However, the structure is anisotropic and its auxetic behavior only appears in two distinct directions.

Since structures with higher symmetry, that is with cubic or isotropic behavior, are of specific interest for researchers and engineers, we use the Zener index $Z=2C_{44}/\left(C_{11}-C_{12}\right)$ (52) to quantify the degree of anisotropy in our structures. It relates the three independent cubic elastic stiffness coefficients and defines the ratio of the maximal and minimal shear modulus of the material. Thus, Z=1 indicates fully isotropic behavior. Fig. 3*C* shows the Poisson’s ratio ν and the Zener ratio for all structures with cubic symmetry, where all possibly isotropic structures lie within the bounds of −1≤ν≤0.5. In addition, no structures with negative Poisson’s ratios are located on or reasonably close to the isotropy line of Z=1. All structures that can be classified as isotropic have Poisson’s ratios of ν≥0.15. However, the catalog contains auxetic structures with cubic symmetry. One example of a novel auxetic structure is the structure cub_Z04.1_E13435 with a Poisson’s ratio of ν=−0.26, shown in the inset z1. In the front view of the structure with 2 × 2 × 2 unit cells in Fig. 3*D*, different patterns that are typical of auxetic structures (51) are identified: the reentrant star, the 3D reentrant triangular structure, and the reentrant pattern. Even though these patterns are frequently found and utilized in literature for the design of cubic auxetic materials, none of the structures from our catalog approaches very low values as reported for example by Chen et al. (20). We attribute this to the fact that in contrast to literature, where extreme values are mostly achieved via complex optimization and search frameworks, our structures are simply derived from crystal networks without any further optimization.

## Extremal Structures

We further search for structures with extremal properties for which the effective stiffness tensor C has one or multiple eigenvalues that are significantly larger than the others (53). The eigenvectors associated with the very small or the relatively large eigenvalues describe the sets of strains under which the structures behave compliant or rigid, respectively. Especially prominent in literature are pentamode structures with five compliant modes and only one rigid mode, which makes them attractive for applications such as elastomechanical cloaking (54). These structures are only rigid under hydrostatic pressure, resulting in a very large bulk modulus B. Ideally, the shear modulus G becomes zero, causing the ratio B/G to become infinitely large. This implies a Poisson’s ratio of $\nu =\frac{3-2\left(G/B\right)}{2\left(G/B\right)+6}=0.5$ and is characteristic for isotropic fluids. Hence, these materials are often referred to as metafluids (14). The previous equation enforces a large ratio B/G for isotropic metafluids as characterized by a Zener index close to 1. The inset at the top right of Fig. 3*C* shows this range of properties in detail. An exemplary structure that qualifies as a quasiisotropic pentamode structure with Z=1.05 and ν=0.496 is the cub_Z03.6_R487 net shown in the inset z2, which describes the molecular arrangement of sodium platinum bronze, ${\text{Na}}_{x}{\text{Pt}}_{3}{\text{O}}_{4}$. The six eigenvalues of the compliance matrix are $\left[9.9\times 10^{-6},\;9.9\times 10^{-6},\;9.9\times 10^{-6},\;1.9\times 10^{-5},\;1.9\times 10^{-5},\;3.3\times 10^{-3}\right],$ where the last eigenvalue is two to three orders of magnitude larger than all other values. The shear modulus $G\approx 9.86\times 10^{-6}$ and the bulk modulus $B\approx 1.11\times 10^{-3}$ yield a ratio of B/G≈113. The eigenvector e≈[−0.58,−0.58,−0.58,0.0,0.0,0.0] associated with the highest eigenvalue represents the three normal stresses and the three shear stresses and confirms that the single rigid mode indeed corresponds to the case of hydrostatic pressure. A similar example from literature in Fig. 3*E* shows the diamond-shaped unit cell as the first manufactured and experimentally validated pentamode metamaterial (14). The biconical shape with a small radius of the bars at the nodes further reduces the shear stiffness of the structure and enables values of $B/G>10^{4}$. To compare the scalability of the pentamode properties of the cub_Z03.6_R487 structure with the results from ref. 55, we replace the cylindrical bars with biconical bars (Fig. 3*F*) and vary the smaller diameter at the nodes d. Fig. 3*G* shows the effective Young’s modulus, shear modulus, and bulk modulus of the cub_Z03.6_R487 structure, obtained by finite-elements simulations using the commercial software package Abaqus 6.14–1 for different values of d on a double-logarithmic scale (details can be found in *SI Appendix*). The results show that the shear modulus and the bulk modulus, like in ref. 55, approximately scale with $G\propto d^{3}$ and B∝d, as indicated by the solid lines. This yields a ratio of B/G≈670 for the smallest d=0.225mm, which is also similar to the results presented in ref. 55 for the structures based on the diamond unit cell. Even though a detailed analysis as in refs. 14, 55 is beyond the scope of this work, this example again shows how the knowledge-based, explorative selection of base cells from our catalog can support researchers to identify and design structures with architected or extremal properties. In general, finding these structures is always a trade-off between efficient numerical modeling to explore the vast design space of cellular structures and comprehensive modeling to fully capture and exploit their complex mechanical and physical behavior. With this work, we lean toward the explorative side of material design and provide a vast number of candidate structures for further exploitation via database-driven design, optimization, and advanced manufacturing methods.

## Conclusion

In summary, we explore the range of mechanical properties of cellular structures, which we obtain by interpreting the spatial arrangement of molecular crystal networks in the context of structural engineering. Our findings are based on the evaluation of 17,087 unique unit cells, which we provide as a ready-to-use catalog, including their geometrical and mechanical description, and visualizations of both. We show that the intrinsic organizing principles of microscopic nets provide a basis for finding well-known and novel cellular structures with tailored properties. We further show that extreme properties often originate from repetitive patterns based on simple geometrical features, which can be related to the underlying crystallographic symmetries. Finally, our work shows that crystallographic knowledge provides a rich source of inspiration for the discovery of novel cellular structures and creates search spaces whose exploration can motivate the design of architected structures with unprecedented properties for future applications across many research fields and length scales.

## Methods

The network descriptions in both crystallographic databases, RCSR and EPINET, are given in a simplified network notation based on lattice unit cells and crystallographic space groups. The space groups provide a comprehensive mathematical description of the symmetries in crystal networks and are categorized with respect to point-symmetry operations and the underlying crystallographic system. To obtain the explicit, unique description of all nodal positions and bar connectivities, we use the software SYSTRE (Symmetry Structure Recognition) (32). SYSTRE uses the relaxed barycentric placement method (33) to compute cells with the ideal symmetry placement of nodes in fractional coordinates and a respective tiling, which are then cropped and converted to Cartesian coordinates (details are provided in *SI Appendix*). The global Cartesian coordinate system orientation is selected for convenience such that the three axes *x*, *y*, *z* coincide with what are the main crystallographic directions of a primitive cubic cell. Following this approach, our catalog of unit cell descriptions is created based on the decoding of a total of 2,730+14,532−135−40=17,087 entries from the RCSR database and the EPINET database, excluding double entries and structures with very small members that cause numerical issues. We apply a numerical homogenization approach (34) based on an Euler–Bernoulli finite-element beam model to all 17,087 unique catalog entries to compute the effective elastic properties of the equivalent cellular structures in form of the symmetric stiffness matrix $C^{\text{H}}$ and the compliance matrix $S^{\text{H}}={\left(C^{\text{H}}\right)}^{-1}$. For comparability, all structures are scaled to a relative density of $\tilde{\rho }=0.01$ at a constant bar radius of r=0.1mm. More details about the implementation can be found in *SI Appendix*. The datasets generated and analyzed during the current study are available in the ETH Zurich Research Collection repository (26, 27).

## Supplementary Material

Supplementary File

Supplementary File

## Data Availability

Data have been deposited in the ETHZ Research Collection at https://doi.org/10.3929/ethz-b-000457598 and https://doi.org/10.3929/ethz-b-000457595. All other study data are included in the article and/or supporting information.
