# Supplementary material for: Exploring the property space of periodic cellular structures based on crystal networks
Source: Proc Natl Acad Sci U S A. 2021 Feb 8;118(7):e2003504118. doi: 10.1073/pnas.2003504118 (PMC7896306; doi:10.1073/pnas.2003504118)
Supplement: Supplementary File [file pnas.2003504118.sapp.pdf]

PNAS
www.pnas.org

Supplementary Information for

Exploring the Property Space of Periodic Cellular Structures Based on Crystal Networks

Thomas S. Lumpe, Tino Stankovic

Corresponding author:
Email: tinos@ethz.ch

This PDF file includes:

- Supplementary text
- Figures S1 to S7
- Tables S1 to S2
- Legend for Video S1
- SI References

Other supplementary materials for this manuscript include the following:

- Video S1

The Unit Cell Catalog

Periodic crystal networks can be described based on a unit cell approach. A unit cell describes the smallest possible basic repeat unit of an infinite lattice with translational symmetry such that any node in $\mathbb{R}^3$ of the lattice can be obtained by a linear combination $a\mathbf{n}_1 + b\mathbf{n}_2 + c\mathbf{n}_3$, where $a, b, c \in \mathbb{Z}$ are multipliers, and $\mathbf{n}_1, \mathbf{n}_2, \mathbf{n}_3 \in \mathbb{R}^3$ are the linearly independent primitive vectors of the lattice with respective lengths of a, b, c. Hence, any crystallographic lattice unit cell is uniquely identified by the lengths of three lattice vectors $\mathbf{a} = a\mathbf{n}_1$, $\mathbf{b} = b\mathbf{n}_2$, $\mathbf{c} = c\mathbf{n}_3$, the angles $\alpha\angle(\mathbf{b}, \mathbf{c})$, $\beta\angle(\mathbf{c}, \mathbf{a})$, and $\gamma\angle(\mathbf{a}, \mathbf{b})$, and a centering that determines the presence of corner nodes, face-centering nodes, and body-centering node. The network descriptions in the two crystallographic databases RCSR (1, 2) and EPINET (3, 4), from which all structures in this work are derived, are given in the simplified network notation based on the lattice unit cells and the symmetry information from the space groups. To obtain the explicit description of all nodal positions and bar connectivities, we use the software SYSTRE (Symmetry Structure Recognition) (5). SYSTRE uses the relaxed barycentric placement method (6) to compute the ideal symmetry placement of nodes and a respective tiling. More concrete, SYSTRE defines the minimal periodic unit U in terms of the nodal positions p_i' in fractional coordinates $u, v, w \in \mathbb{R}$ for all nodes $i = 1 \dots n$ in U, as well as the connectivity $e_k = \left\{\{p_i', p_j'\} | p_i', p_j' \in U, i \neq j\right\}$ between two nodes p_i' and p_j' for all bars $k = 1 \dots m$ of the minimal periodic unit. However, because of the crystallographic tiling procedure this minimal unit not necessarily coincides with the lattice unit cell boundaries of $[0, 0, 0] \leq [u, v, w] \leq [1, 1, 1]$. Bars often cross the unit cell boundaries and partly lie in adjacent unit cells. To decode the crystallographic information and generate unit cells as used in the engineering context, we first tessellate the minimal periodic unit cell to obtain a structure that is 3x3x3 unit-cells in size. Next, we cut out the center cell to obtain all bars lying inside and on the boundary of the lattice unit cell, thus obtaining the unit cell of a cellular structure with full prescribed symmetry. Hence, bars that originally penetrate the unit cell boundary are cut off and only the part inside of the unit cell is retained. Depending on the actual topology of the structure, this can lead to unconnected bars in the unit cell. Due to the translational symmetry, the full bars are recovered when the single unit cell is tessellated along the lattice vectors $\mathbf{a}, \mathbf{b}, \mathbf{c}$. Finally, the nodal positions in Cartesian coordinates $p(x, y, z)$ are retrieved via

$$\begin{bmatrix} x \\ y \\ z \end{bmatrix} = \begin{bmatrix} a & b\cos(\gamma) & c\cos(\gamma) \\ 0 & b\sin(\gamma) & c\,\frac{\cos(\alpha)-\cos(\beta)\cos(\gamma)}{\sin(\gamma)} \\ 0 & 0 & \frac{\Omega}{ab\sin(\gamma)} \end{bmatrix} \begin{bmatrix} u \\ v \\ w \end{bmatrix},$$

with the volume Ω of the parallelepiped spanned by $(\mathbf{a}, \mathbf{b}, \mathbf{c})$

$$\Omega = abc\sqrt{1 - \cos^2(\alpha) - \cos^2(\beta) - \cos^2(\gamma) + 2\cos(\alpha)\cos(\beta)\cos(\gamma)}.$$

The global Cartesian coordinate system orientation is selected for convenience such that the three axes *x, y, z* coincide with what are the main crystallographic directions of a primitive cubic cell. Due to the relaxed barycentric placement method used in SYSTRE, some structures have overlapping or intersecting bars. These structures are marked as such in the unit cell catalog (7).

Following this approach, a catalog of unit cell descriptions is created based on the decoding of in total $2{,}730 + 14{,}532 = 17{,}262$ entries from the RCSR database and the EPINET database. After excluding 135 duplicate entries and 40 structures with very small members that cause numerical inaccuracies, 17,087 unique structures are analyzed and discussed in this work. For completeness, all duplicate entries and entries with very small members are also provided in the catalog and the structures are marked respectively. In (7), we provide the full text-based unit cell catalog, which includes for all structures: names, normalized unit cell parameters, average connectivity, effective mechanical properties, maximum overall stiffness, stiffness scaling parameters, nodal positions, and bar connectivities. Reference (8) includes images of all structures and 360° elastic surface plots of the orientation-dependent Young's modulus. The Young's modulus in an arbitrary direction E' is computed according to (9) as $E' = 1/s'$ where $s' = l_i l_j l_m l_n S_{ijmn}$, l_i are guiding cosines corresponding to a spherical coordinate system and S_{ijmn} is the compliance tensor.

Numerical Homogenization

To compute the effective properties of all cellular structures in the catalog in form of the symmetric stiffness matrix $\mathbf{C}^{\mathrm{H}}$ and the compliance matrix $\mathbf{S}^{\mathrm{H}} = (\mathbf{C}^{\mathrm{H}})^{-1}$, we use the numerical homogenization approach described in (10), based on an in-house Euler-Bernoulli beam FEM code written in MATLAB. The effective stiffness tensor of a discretized periodic cellular structure can be computed based on a unit cell as

$$\mathbf{C}^{\mathrm{H}} = \frac{1}{|Y|}\left(\boldsymbol{\chi}^{0(ij)} - \tilde{\boldsymbol{\chi}}^{ij}\right)^{\mathrm{T}}\left(\mathbf{f}^{kl} - \mathbf{f}^{*(kl)}\right)\mathrm{d}Y,$$

where Y is the domain of the unit cell, $\boldsymbol{\chi}$ are the nodal displacements in the unit cell, and $\mathbf{f}$ are the internal forces in the unit cell. $\boldsymbol{\chi}^{0(ij)}$ represents the unit displacements that are initially applied to the unit cell. For the general anisotropic 3D case, six different load cases are required, and the respective displacement fields in terms of the global *x, y, z* coordinates are obtained via

$$\boldsymbol{\chi}^{0(11)} = \begin{Bmatrix} x \\ 0 \\ 0 \end{Bmatrix}, \boldsymbol{\chi}^{0(22)} = \begin{Bmatrix} 0 \\ y \\ 0 \end{Bmatrix}, \boldsymbol{\chi}^{0(33)} = \begin{Bmatrix} 0 \\ 0 \\ z \end{Bmatrix}$$

$$\boldsymbol{\chi}^{0(12)} = \boldsymbol{\chi}^{0(21)} = \begin{Bmatrix} 0.5y \\ 0.5x \\ 0 \end{Bmatrix}, \boldsymbol{\chi}^{0(13)} = \boldsymbol{\chi}^{0(31)} = \begin{Bmatrix} 0.5z \\ 0 \\ 0.5x \end{Bmatrix}, \boldsymbol{\chi}^{0(23)} = \boldsymbol{\chi}^{0(32)} = \begin{Bmatrix} 0 \\ 0.5z \\ 0.5y \end{Bmatrix}.$$

The resulting nodal forces can be calculated by $\mathbf{f}^{ij} = \mathbf{K}\boldsymbol{\chi}^{0(ij)}$, with the stiffness matrix $\mathbf{K}$ of the discretized unit cell. To obtain the displacements $\tilde{\boldsymbol{\chi}}^{ij}$, the forces $\mathbf{f}^{ij}$ are applied to the same unit cell under periodic boundary conditions $\tilde{\boldsymbol{\chi}}^{ij} = \tilde{\mathbf{K}}^{-1}\mathbf{f}^{ij}$. Due to the periodicity of the unit cells in the catalog, periodic boundary conditions can be specified to nodes on opposing faces and edges. Mathematically, this is achieved by a matrix multiplication that relates a vector $\mathbf{u}$ with full n degrees of freedom to its reduced version $\tilde{\mathbf{u}}$ via the transformation matrix $\mathbf{T}$ with $\mathbf{u} = \mathbf{T}\tilde{\mathbf{u}}$ (11). For m coupled degrees of freedom, $\tilde{\mathbf{u}}$ has the size $(n - m)$ and $\mathbf{T}$ has the size $n \times (n - m)$. In this case, the degrees of freedom of the nodes on the six faces *left, right, bottom, top, front, back*, on the twelve edges *E1-E12*, and on the eight vertices *1-8* of the unit cell are coupled to the master degrees of freedom on the faces *left, bottom, front*, on the edges *E1, E5, E9*, and on the vertex *1*:

$$\begin{bmatrix} p_{left} \\ p_{right} \\ p_{bottom} \\ p_{top} \\ p_{front} \\ p_{back} \\ p_{\mathrm{E1}} \\ p_{\mathrm{E2}} \\ p_{\mathrm{E3}} \\ p_{\mathrm{E4}} \\ p_{\mathrm{E5}} \\ p_{\mathrm{E6}} \\ p_{\mathrm{E7}} \\ p_{\mathrm{E8}} \\ p_{\mathrm{E9}} \\ p_{\mathrm{E10}} \\ p_{\mathrm{E11}} \\ p_{\mathrm{E12}} \\ p_1 \\ p_2 \\ p_3 \\ p_4 \\ p_5 \\ p_6 \\ p_7 \\ p_8 \end{bmatrix} = \begin{bmatrix} \mathbf{I} & \mathbf{0} & \mathbf{0} & \mathbf{0} & \mathbf{0} & \mathbf{0} & \mathbf{0} \\ \mathbf{I} & \mathbf{0} & \mathbf{0} & \mathbf{0} & \mathbf{0} & \mathbf{0} & \mathbf{0} \\ \mathbf{0} & \mathbf{I} & \mathbf{0} & \mathbf{0} & \mathbf{0} & \mathbf{0} & \mathbf{0} \\ \mathbf{0} & \mathbf{I} & \mathbf{0} & \mathbf{0} & \mathbf{0} & \mathbf{0} & \mathbf{0} \\ \mathbf{0} & \mathbf{0} & \mathbf{I} & \mathbf{0} & \mathbf{0} & \mathbf{0} & \mathbf{0} \\ \mathbf{0} & \mathbf{0} & \mathbf{I} & \mathbf{0} & \mathbf{0} & \mathbf{0} & \mathbf{0} \\ \mathbf{0} & \mathbf{0} & \mathbf{0} & \mathbf{I} & \mathbf{0} & \mathbf{0} & \mathbf{0} \\ \mathbf{0} & \mathbf{0} & \mathbf{0} & \mathbf{I} & \mathbf{0} & \mathbf{0} & \mathbf{0} \\ \mathbf{0} & \mathbf{0} & \mathbf{0} & \mathbf{I} & \mathbf{0} & \mathbf{0} & \mathbf{0} \\ \mathbf{0} & \mathbf{0} & \mathbf{0} & \mathbf{I} & \mathbf{0} & \mathbf{0} & \mathbf{0} \\ \mathbf{0} & \mathbf{0} & \mathbf{0} & \mathbf{0} & \mathbf{I} & \mathbf{0} & \mathbf{0} \\ \mathbf{0} & \mathbf{0} & \mathbf{0} & \mathbf{0} & \mathbf{I} & \mathbf{0} & \mathbf{0} \\ \mathbf{0} & \mathbf{0} & \mathbf{0} & \mathbf{0} & \mathbf{I} & \mathbf{0} & \mathbf{0} \\ \mathbf{0} & \mathbf{0} & \mathbf{0} & \mathbf{0} & \mathbf{I} & \mathbf{0} & \mathbf{0} \\ \mathbf{0} & \mathbf{0} & \mathbf{0} & \mathbf{0} & \mathbf{0} & \mathbf{I} & \mathbf{0} \\ \mathbf{0} & \mathbf{0} & \mathbf{0} & \mathbf{0} & \mathbf{0} & \mathbf{I} & \mathbf{0} \\ \mathbf{0} & \mathbf{0} & \mathbf{0} & \mathbf{0} & \mathbf{0} & \mathbf{I} & \mathbf{0} \\ \mathbf{0} & \mathbf{0} & \mathbf{0} & \mathbf{0} & \mathbf{0} & \mathbf{I} & \mathbf{0} \\ \mathbf{0} & \mathbf{0} & \mathbf{0} & \mathbf{0} & \mathbf{0} & \mathbf{0} & \mathbf{I} \\ \mathbf{0} & \mathbf{0} & \mathbf{0} & \mathbf{0} & \mathbf{0} & \mathbf{0} & \mathbf{I} \\ \mathbf{0} & \mathbf{0} & \mathbf{0} & \mathbf{0} & \mathbf{0} & \mathbf{0} & \mathbf{I} \\ \mathbf{0} & \mathbf{0} & \mathbf{0} & \mathbf{0} & \mathbf{0} & \mathbf{0} & \mathbf{I} \\ \mathbf{0} & \mathbf{0} & \mathbf{0} & \mathbf{0} & \mathbf{0} & \mathbf{0} & \mathbf{I} \\ \mathbf{0} & \mathbf{0} & \mathbf{0} & \mathbf{0} & \mathbf{0} & \mathbf{0} & \mathbf{I} \\ \mathbf{0} & \mathbf{0} & \mathbf{0} & \mathbf{0} & \mathbf{0} & \mathbf{0} & \mathbf{I} \\ \mathbf{0} & \mathbf{0} & \mathbf{0} & \mathbf{0} & \mathbf{0} & \mathbf{0} & \mathbf{I} \end{bmatrix} \begin{bmatrix} p_{\mathrm{left}} \\ p_{\mathrm{bottom}} \\ p_{\mathrm{front}} \\ p_{\mathrm{E1}} \\ p_{\mathrm{E5}} \\ p_{\mathrm{E9}} \\ p_1 \end{bmatrix}.$$

To obtain $\mathbf{f}^{*(ij)}$, the periodic displacements $\tilde{\boldsymbol{\chi}}^{ij}$ are applied to the original unit cell without periodic boundary conditions as $\mathbf{f}^{*(ij)} = \mathbf{K}\tilde{\boldsymbol{\chi}}^{ij}$.

For comparability, all structures are scaled to a relative density of $\rho = 0.01$ at a constant bar radius of $r = 0.1\ \mathrm{mm}$. The volume of the structures is calculated by summing up the volume of all bars, where bars on the edges or faces of the unit cell only contribute partially. The most relevant engineering constants for all structures, i.e. the effective generalized Young's moduli, shear moduli and Poisson's ratios, are determined in the global *x-, y-, z*-directions as

$$E_x = \frac{1}{S_{11}}, E_y = \frac{1}{S_{22}}, E_z = \frac{1}{S_{33}}, G_{yz} = \frac{1}{S_{44}}, G_{xz} = \frac{1}{S_{55}}, G_{xy} = \frac{1}{S_{66}}$$

$$\nu_{yz} = -S_{23} * E_y, \nu_{xz} = -S_{13} * E_x, \nu_{xy} = -S_{12} * E_x,$$

$$\nu_{zy} = -S_{23} * E_z, \nu_{zx} = -S_{13} * E_z, \nu_{yx} = -S_{12} * E_y,$$

with the effective Young's moduli E_i, the effective shear moduli G_{ij} and the Poisson's ratios ν_{ij}. For structures with cubic symmetry, the bulk modulus is calculated via $B = \frac{1}{3}(C_{11} + 2C_{12})$. (12)

Properties of Pentamode Structures

To numerically estimate the scalability of the pentamode properties of the cub_Z03.6_R487 structure (Fig. 3F in the main manuscript) and compare it with a pentamode structure from literature, we compute the mechanical properties of the cub_Z03.6_R487 structure with finite elements simulations using the commercial software package Abaqus 6.14-1 Standard. The cylindrical bars are replaced with biconical bars, with the values of the bar diameter at the nodes $d \in [0.225 \text{ mm}, 1.305 \text{ mm}]$, the maximum diameter in the middle of the bars $D = 1.305 \text{ mm}$, and the unit cell size $a = 15 \text{ mm}$. We choose a linear-elastic material model with the base material's Young's modulus $E_{\text{m}} = 1.4 \text{ GPa}$, and the base material's Poisson's ratio $\nu_{\text{m}} = 0.4$. Every model is discretized using quadratic C3D10 tetrahedral elements. To reduce the effects of possible anisotropies on the results, we obtain the bulk modulus simply by its definition, i.e. by applying hydrostatic pressure to the structure and calculating its change in volume. The Young's modulus and the shear modulus are determined by applying a standard compression and shear load to the structure, respectively, and computing the resulting deformations.

Validation of Numerical Homogenization Results

To verify the mechanical properties obtained by our numerical homogenization framework, we compare our results to analytical results from literature and to experimental results. For low relative densities of about $\bar{\rho} < 0.1$, the stiffness of structures can be described by classical scaling laws of the form $E/E_{\text{s}} = C * \bar{\rho}^n$, with the effective Young's modulus E, the Young's modulus of the solid base material E_{s}, a scaling parameter C, and a scaling exponent n (13). A scaling exponent of $n = 1$ indicates stretch-dominated behavior, whereas $n = 2$ describes structures with bending-dominated behavior.

To compare and verify the properties of different structures from the catalog, Fig. S1 shows the normalized Young's modulus E/E_{s} of the four structures cub_Z14.0_E38 (reinforced body-centered), cub_Z12.0_E19 (octet-truss), cub_Z04.0_E970 (Kelvin-cell or tetrakaidecahedron), and cub_Z08.0_E3 (body-centered cubic) at low relative densities between $0.005 \leq \bar{\rho} \leq 0.1$. The solid dots show the results obtained with our homogenization framework. The mechanical properties of the octet-truss and the Kelvin cell were in-depth investigated analytically in literature (14, 15) and the respective scaling relations are given by the dash-dotted lines. The dashed line shows the Voigt bound as the maximum attainable stiffness of a general cellular structure for a given relative density (16). The results from our homogenization framework show very good agreement with the analytical results and confirm the validity for structures with relative densities $\bar{\rho} < 0.1$. For the reinforced body-centered cubic and the body-centered cubic structure, the solid lines indicate a power fit of the homogenization results to determine the parameters C and n of the classical scaling law. The fit yields scaling exponents of $n = 1.00$ (reinforced body-centered cubic) and $n = 2.00$ (body-centered cubic) and confirms the expected stretch- and bending-dominated behavior, respectively.

To further verify the numerical results, we 3D printed 2x2x2 unit cells of all four structures, conducted uniaxial compression tests, and compare the experimentally obtained stiffness to the analytical and numerical results. All test specimens were printed on a Stratasys Connex3 Objet500 3D printer (Stratasys Ltd., Eden Prairie, MN, USA, and Rehovot, Israel) using the PolyJet material jetting technology. Standard printing parameters were used, with the "matte" option to fully cover the structures in support material. The support material was removed with a water jet machine. We fabricated a set of five structures for each cell type with effective strut diameters between 1.7 mm and 3.0 mm and overall edge lengths of the structures of about 66mm (reinforced-body centered), 75 mm (octet-truss), 54 mm (Kelvin-cell), and 68 mm (BCU).

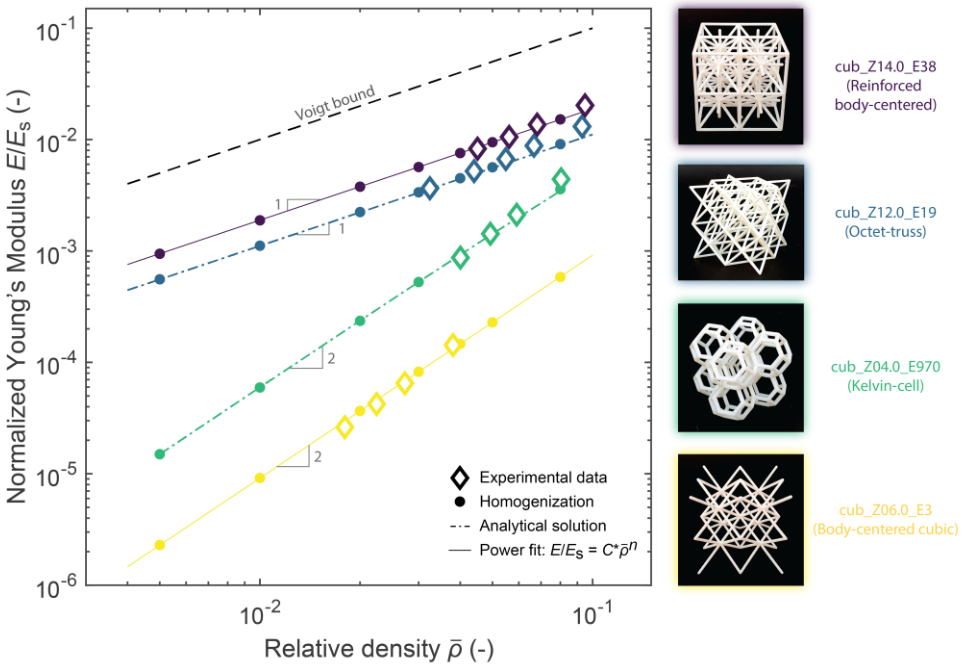

Fig. S1. Scaling behavior for relative densities below 0.1 of the four cell types reinforced body-centered (purple), octet-truss (blue), Kelvin-cell (turquoise), and body-centered cubic (yellow). The solid dots show results obtained via our numerical homogenization framework. The dash-dotted lines show analytical scaling relations from literature (14, 15), and the solid lines indicate an power fit of the form $E/E_S = C * \bar{\rho}^n$. The diamond markers show experimental results from uniaxial compression tests.

For all cell types except for the octet-truss, one structure was discarded due to printing errors. Additionally, solid cylindrical columns with a diameter of 15 mm and a height of 30 mm were printed with the same printing parameters. We performed uniaxial compression tests with an Instron ElectroPuls E3000 testing machine, a Dynacell load cell of 5kN capacity, and a strain rate of $\dot{\epsilon} = 10^{-3}$. The slope of the linear regime of the stress-strain curve was used to compute the effective Young's modulus E and E_S of the structures and of the solid base material, respectively. The experimental results are indicated by the diamond markers in Fig. S1 and show generally good agreement with the theoretical results at lower densities $\bar{\rho} < 0.07$, but show stiffer behavior close to $\bar{\rho} = 0.1$. This is in agreement with findings from literature, where the increase in stiffness is explained by the increasing influence of the nodes at higher relative densities (13).

In summary, analytical and experimental results confirm the validity of our homogenization approach for structures with low relative densities of $\bar{\rho} < 0.07$. Since we limit this paper to structures at relative densities of 1%, we can conclude that our results are accurate within the limits of linear elasticity.

Crystallographic Symmetries

The structures in the catalog can be classified according to their space group, which in turn can be summarized with respect to the crystal systems, i.e. Triclinic, Monoclinic, Orthorhombic, Tetragonal, Trigonal, Hexagonal, and Cubic. Fig. S2 shows the number of structures in the catalog of each crystal system on a logarithmic scale. Tetragonal, orthorhombic, and cubic structures are found the most, whereas the catalog contains only three triclinic structures. Since the crystal system is linked to the underlying symmetry of the structure, it directly influences the topological design and especially the mechanical properties of a structure (Neumann's principle (17)).

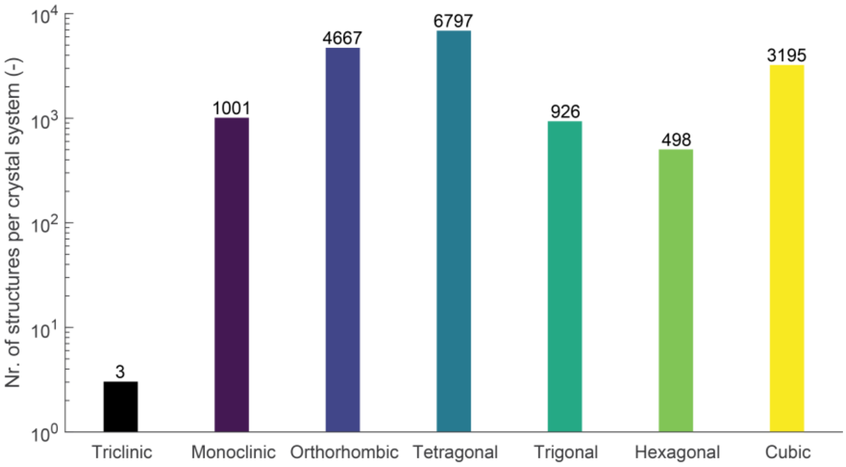

Fig. S2. Number of unique structures per crystal system.

Triclinic structures have the lowest symmetry, i.e. at most an inversion center at the coordinate origin. Monoclinic structures have a unique 2-fold axis of rotation, orthorhombic structures have three mutually perpendicular 2-fold axes of rotation. The tetragonal system has a unique 4-fold axis of rotation, which coincides with the *z*-direction. Trigonal and hexagonal structures have a unique 3-fold or 6-fold axis of rotation, respectively, which also coincide with the z-direction. The cubic system as the system with the highest symmetry has three axes of rotation at right angles to each other. (18)

Stiffness Anisotropy

Fig. S3 shows the effect of the crystal system on the anisotropy of the stiffness, i.e. the direction-dependence of the Young's modulus. Due to the low number of structures (three in total), the triclinic crystal system is omitted. Fig. S3*A* shows the percentage of all structures per crystal system that show maximum uniaxial stiffness (i.e. maximum Young's modulus) in the direction of at least one of the main coordinate system axes, i.e. along the *x*-direction, the *y*-direction, or the *z*-direction. Fig. S3*B* further shows along which of these three main directions the maximum stiffness occurs. Since structures can have the stiffest direction along only one, two, or along all three directions simultaneously (Fig. S3*B*), the bars do not always add up to the values from Fig. S3A.

Since there is only a single two-fold rotational symmetry element for all monoclinic structures, structural features in one direction are often not replicated in orthogonal directions and remain unique. Hence, structures with maximum stiffness in one direction will most likely have only this single stiffest direction. The same holds for orthorhombic structures with two-fold rotation axes. In contrast to the monoclinic system, orthorhombic unit cells however have a rectangular unit cell with $\alpha = \beta = \gamma = 90°$, which makes the direction of its base vectors coincide with the cartesian coordinate directions. Hence, structures with maximum stiffness in these directions are more likely than in the monoclinic system. With a fixed axis of rotation in the *z*-direction, tetragonal, trigonal, and hexagonal systems include many structures where the stiffest direction coincides with the *z*-axis. The cubic system with the highest symmetry ensures that if a structure has maximum stiffness in one direction, it is equally stiff in the two orthogonal directions.

Even though Fig. S3 and the 360° elastic surface plots in (8) only describe the anisotropy of the effective Young's modulus, they can also provide information about the respective shear moduli. The structure cub_Z07.4_R679 presented in the main manuscript has high shear stiffness G_{xz}, G_{yx}, and G_{xy}. The 360° plot of its Young's modulus shows a strong anisotropy, where the stiffest directions are the 45° axes with respect to the main coordinate system directions. This is indicated by "spikes" in these directions, whereas the stiffness in all other directions is orders of magnitude lower. In contrast to that, structures

such as cub_Z06.0_E1 have a high Young's modulus only in the *x-, y-, z*-directions, and consequently have very low values of G_{xz}, G_{yx}, and G_{xy}. For quasi-isotropic structures, where the 360° plot shows an almost perfect sphere, the effective properties are described by only two independent parameters and are linked e.g. by $G = E/(2*(1+\nu))$. All structures where the stiffest direction does not coincide with the global *x*-, *y*-, or *z*-direction are marked in the unit cell catalog (7) and the overall maximum stiffness values are given. The direction(s) of the maximum stiffness can be visually identified from the 360° elastic surfaces in (8).

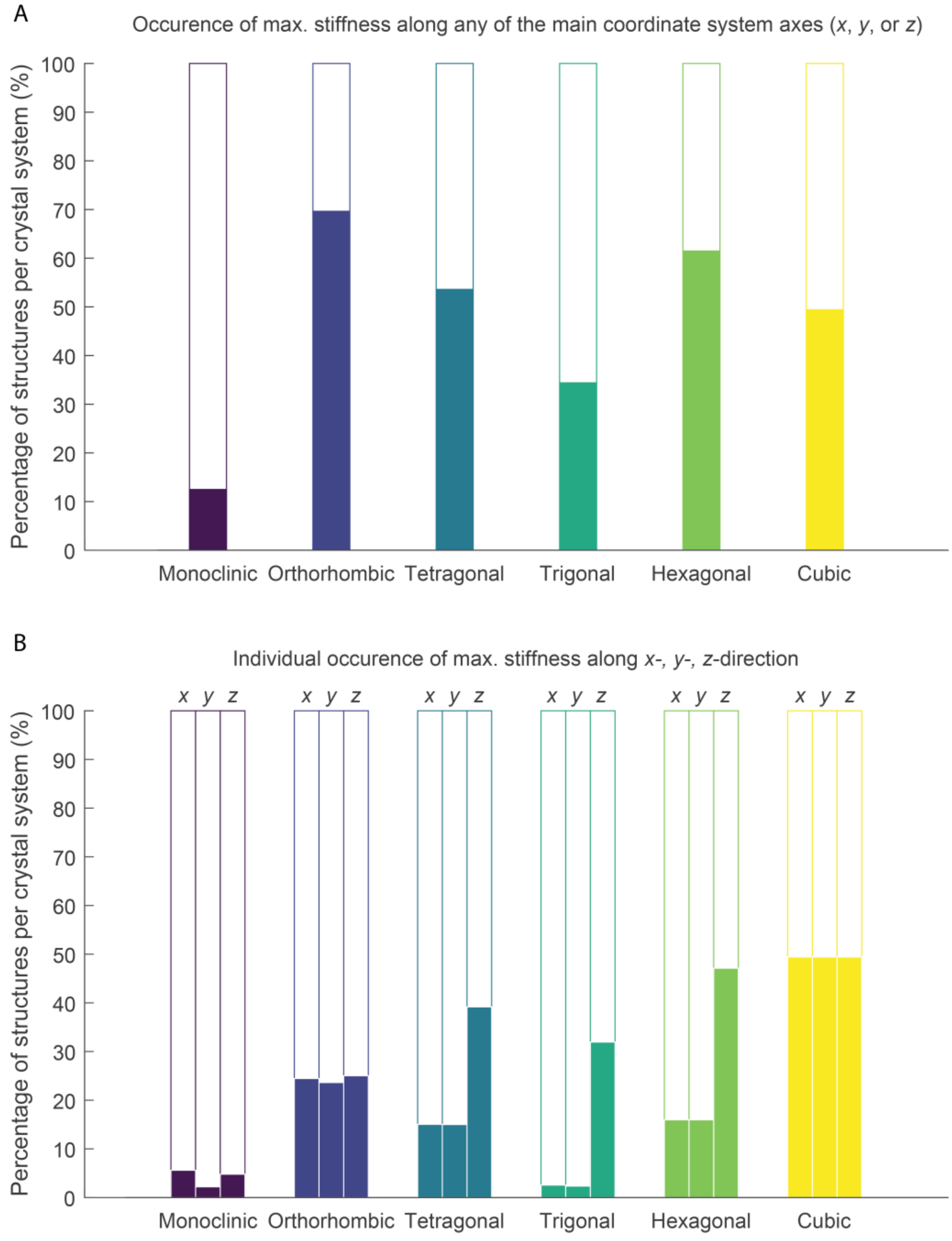

Fig. S3. (*A)* Percentage of structures per crystal system with maximum uniaxial stiffness, i.e. maximum effective Young's modulus, either along the *x*-direction, *y*-direction, or *z*-direction of the global Cartesian coordinate system. *(B)* Percentage of structures per crystal system with max. stiffness along each of these axes individually.

Poisson’s Ratio

The effect of crystallographic symmetries on the Poisson’s ratio is discussed in the main text for the directions *zx*/*zy*. For completeness, we provide the plots for the remaining directions *xy*/*xz* and *yx*/*yz* here (Fig. S4*A* and Fig. S4*B*)

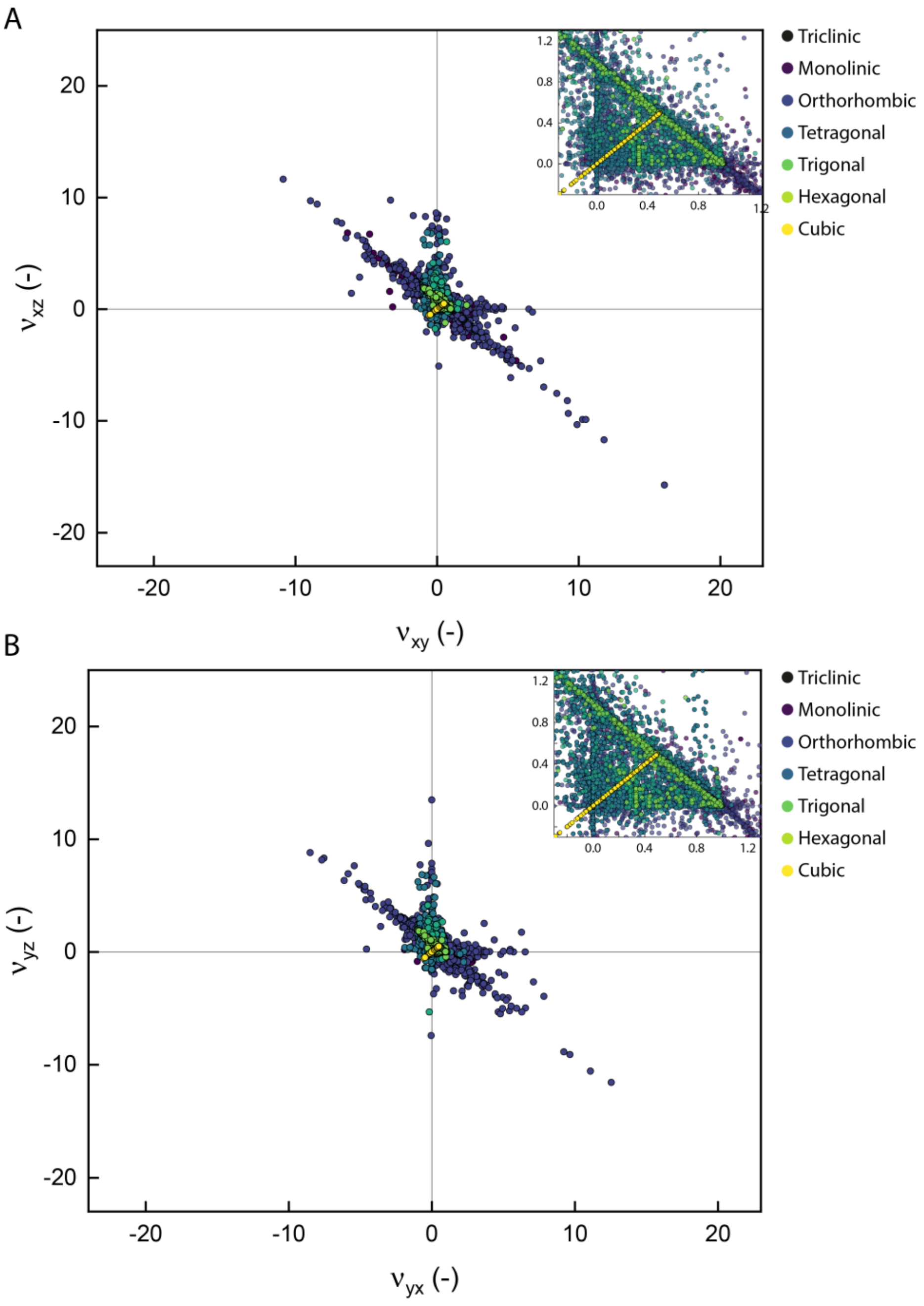

Fig. S4. Poisson’s ratios (*A*) ν_{xy}, ν_{xz} and (*B*) ν_{yx}, ν_{yz} of all structures in the catalog. The colors indicate the crystal system of the structures.

Stiffness Scaling and Connectivity

In this paper, we mostly cover the mechanical properties of cellular structure at a relative density of 0.01. Since these properties can strongly vary at different relative densities, scaling laws can help to compare different structures. Fig. S5 shows the scaling parameter C and the scaling exponent n of all structures in the catalog of the elastic directional stiffness in *x*-direction, *y*-direction, and *z*-direction. These parameters represent the classical stiffness scaling relation for lattice structures, which is given by $E/E_s = C * \bar{\rho}^n$ (e.g. (13, 19)). The parameter n indicates the mechanical behavior of cellular structures. For stretch-dominated behavior, $n = 1$, and for bending-dominated behavior, $n = 2$. The parameters are computed by applying our numerical homogenization framework to all structures in the catalog at five relative densities between 0.005 and 0.01 and applying a power fit to the resulting linear-elastic Young's moduli in the three main coordinate system directions *x, y, z*. For all structures, we obtain R^2 values strictly greater than 0.996. Histograms show the occurrence of specific parameter values in the catalog. The scaling exponents n_i of all structures in all three directions (where *i* denotes the *x*-, *y*-, *z*-direction) lie roughly between 1 and 2. For about 60% of all structures, n is smaller than 1.02 or larger than 1.98, indicating pure stretch- or bending-dominated behavior in the respective directions. The scaling constant C determines how fast the stiffness increases with increasing relative density and lies for most structures between 0 and 1. For comparability, all values are provided in the unit cell catalog (7).

The connectivity at the nodes of a cellular structure is often used to describe the rigidity and hence the mechanical behavior of a structure. Deshpande et al. (20) showed that an average connectivity (number of struts at a node) of $Z_a = 6$ is a necessary, but not sufficient condition for the rigidity of 3D frameworks. Fig. S6 confirms that this condition is not sufficient, since many structures with non-rigid, non-stretch-dominated behavior ($n \geq 1$) can be found for values of $Z_a \geq 6$. The opposite does not hold, i.e. structures with $n = 1$ are not always fully stretch-dominated. For example, overall bending-dominated structures such as the simple cubic structure cub_Z06.0_E1 can show stretch-dominated behavior along certain directions (here e.g. in the direction of the bars which are oriented along the global *x-, y-, z*-directions). For structures with similarly situated nodes, i.e. for nodes where the rest of the structure appears the same and in the same orientation if viewed from any of the nodes, Deshpande et al. (20) find $Z_a \geq 12$ a sufficient condition for the rigidity of periodic 3D frameworks. Indeed, no similarly situated structures with overall non-stretch-dominated behavior ($n_{x,y,z} > 1$) and $Z_a \geq 12$ are found in the catalog. As a reference, the position of the well-known octet-truss structure (cub_Z12.0_E19) that is similarly situated and fulfills the necessary and sufficient criteria ($Z_a = 12$), is marked in Fig. S6. The average connectivity values of all structures are also provided in the unit cell catalog (7).

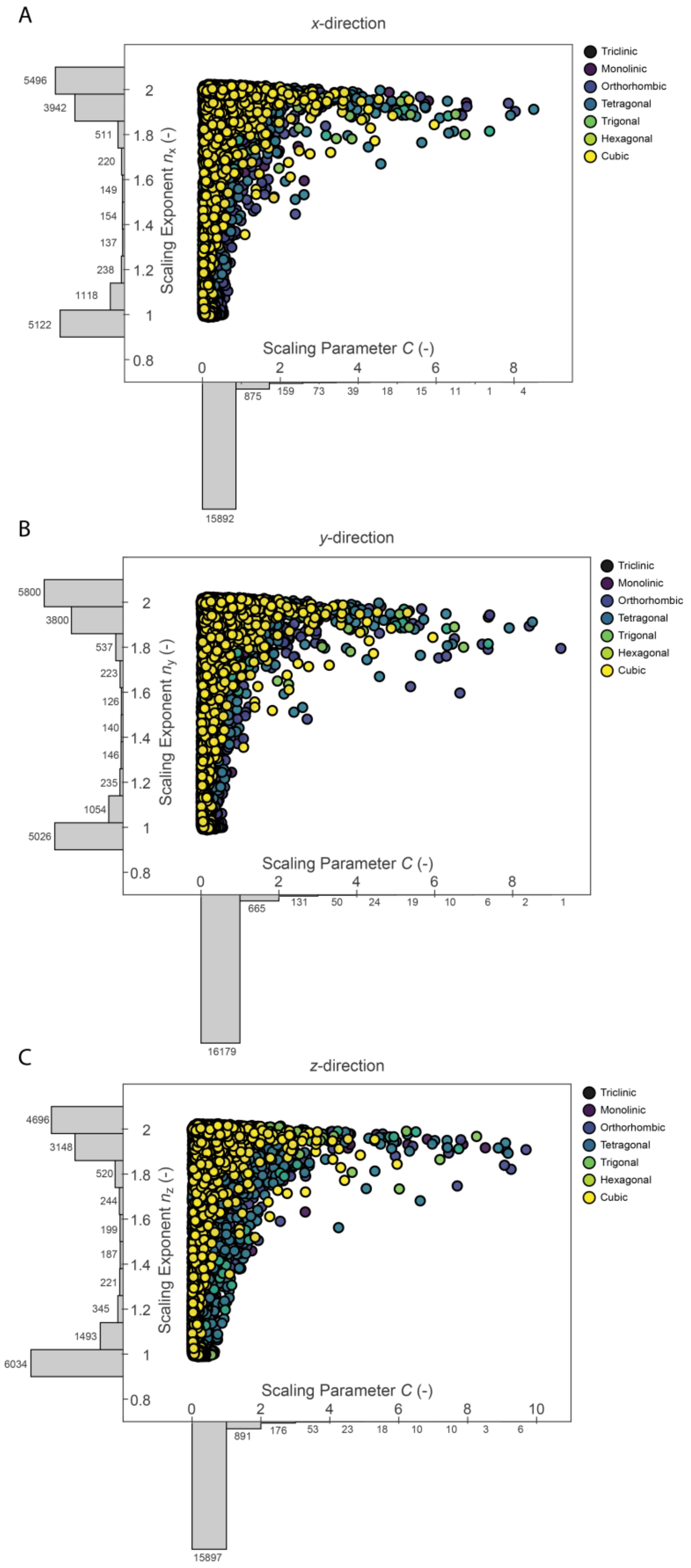

Fig. S5. Scaling parameters C and scaling exponents n in *(A) x*-direction, *(B) y*-direction, and *(C) z*-direction.

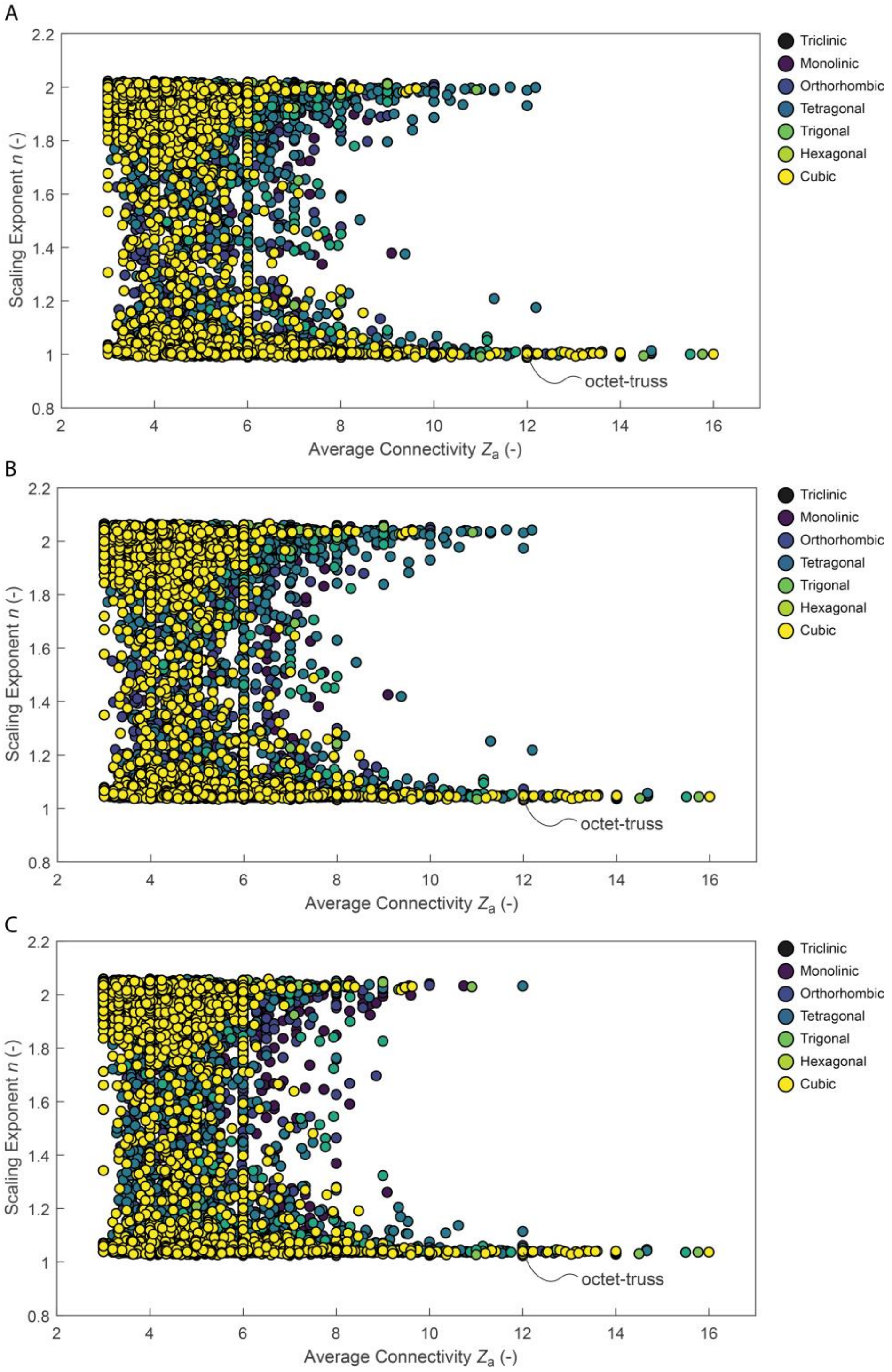

Fig. S6. Average connectivity Z_a and scaling exponents n in *(A) x*-direction, *(B) y*-direction, and *(C) z*-direction.

Theoretical Bounds of Cellular Structures

For a general anisotropic cellular structure, the Voigt bound determines the theoretical maximum stiffness (16). As a function of the constituent materials' elastic moduli and the relative density, the Voigt bound on the Young's modulus E and on the shear modulus G are computed as $E_{\mathrm{Voigt}} = E_{\mathrm{s}} * \bar{\rho}$ and $G_{\mathrm{Voigt}} = G_{\mathrm{s}} * \bar{\rho}$, respectively. For example, the maximum effective Young's modulus of a cellular structure with 10% relative density can be 10% of the base material's Young's modulus at maximum. Fig. S7*A* shows the effective Young's modulus E_x and the effective shear modulus G_{yz} of all structures in the catalog. The Voigt bounds are indicated by the dotted lines. The insets v1 to v3 show structures close to the bounds. The topological and geometrical patterns found in these structures can directly be related to their mechanical behavior and are representative of architectures in the vicinity of the points, respectively. The structure ort_Z04.0_E28 in inset v1 has a high stiffness in *x*-direction, but a very low shear stiffness in the *yz*-plane. The multiple straight bars in *x*-direction make it very stiff, while the single bar in *z*-direction provides very little resistance against shear deformations. Bars parallel to the main coordinate system directions are also identified as geometries with high stiffness in the main manuscript. The structure ort_Z04.3_E1408 in inset v2 has both high stiffness in *x*-direction and high shear stiffness in the *yz*-plane. The high unidirectional stiffness is again caused by straight bars, and the additional diagonal bars at an angle of 45° with respect to the *y*-axis provide high shear stiffness. High shear stiffness caused by diagonal bars is also found in the structure ort_Z04.7_R1673 in inset v3. However, this structure has no bars parallel to any of the main coordinate system directions and low average connectivity of 4.7, and is hence very compliant under compressive loads in any of these directions. These patterns identified are very common with structures close to the Voigt bounds, but often occur in anisotropic structures with low symmetry.

Since structures with isotropic mechanical properties are of special interest for many applications due to their direction-independent behavior, we further consider their mechanical properties with respect to more specific theoretical bounds. The Hashin-Shtrikman bounds describe the upper and lower bounds for the effective elastic moduli of quasi-isotropic multi-phase structures and materials. They provide a much closer estimate of the bounds of a structure than the more general Voigt bound, which is independent of the structural symmetries. The upper Hashin-Shtrikman bounds on the bulk and shear modulus of quasi-isotropic materials are given by (21)

$$B^* = B_2 + \frac{\phi_1}{\frac{1}{B_1 - B_2} + \frac{3\phi_2}{3B_2 + 4G_2}}$$

$$G^* = G_2 + \frac{\phi_1}{\frac{1}{G_1 - G_2} + \frac{6(B_2 + 2G_2)\phi_2}{5G_2(3B_2 + 4G_2)}}$$

with the base bulk moduli $B_2 > B_1$, the base shear moduli $G_2 > G_1$, and the volume fractions of both base materials ϕ_1, ϕ_2. Here, $B_1 = G_1 = 0$ represent the void phase, and $B_2 = B_{\mathrm{s}}$, $G_2 = G_{\mathrm{s}}$ indicate the properties of the solid base material. Figure S7*B* shows the effective bulk and shear modulus of the 232 structures in the catalog with cubic symmetry and a Zener-ratio of $0.9 \leq Z \leq 1.1$, i.e. structures with close to isotropic properties. The dashed line and the dotted line show the upper Hashin-Shtrikman bounds and the Voigt bounds, respectively. Even though our catalog provides a large variety of different structures, no structure approaches the theoretical limits, as partially achieved by structures from literature (e.g. (22)). We attribute this to the fact that in literature, the bounds are attained by structures that result from complex optimization frameworks, whereas our cells are simply generated from crystal networks without being optimized for any objective at all.

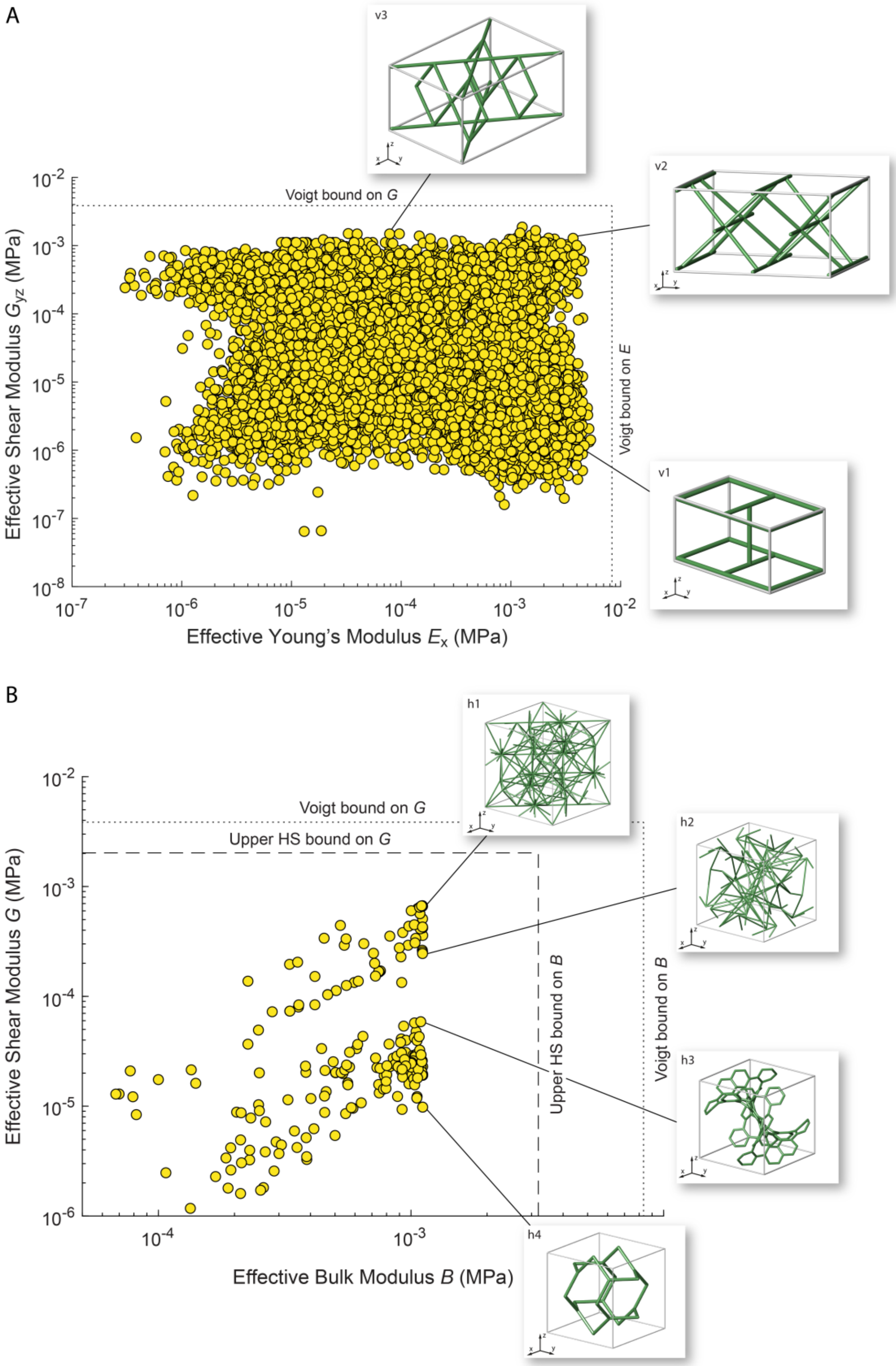

Fig. S7. (*A*) Effective Young's modulus E_x and shear modulus G_{yz} of all structures in the catalog. The dotted lines indicate the Voigt bounds on the bulk and shear modulus. (*B*) Effective bulk and shear moduli of the 232 structures with cubic symmetry and a Zener-ratio of $0.9 \leq Z \leq 1.1$, i.e. structures with close to isotropic properties. The dashed lines indicate the upper Hashin-Shtrikman bounds on the bulk and shear modulus. The dotted lines indicate the Voigt bounds on the bulk and shear modulus.

Examples of structures closest to the bounds are shown in the insets h1 to h4 and are representative of architectures in the neighbourhood of the points, respectively. The insets h1 and h2 show the structures cub_Z12.8_R970 and cub_Z08.0_R1966 with high shear modulus and high bulk modulus. Both structures have relatively high average connectivities, which result in high overall stiffness. Simple patterns of straight bars only in the main coordinate system directions are not found anymore, since isotropic material behavior requires equal stiffness in all directions. The insets h3 and h4 show the structures cub_Z03.0_R1339 and cub_Z03.6_R487, which both have a high bulk modulus but relatively low shear stiffness. In contrast to the structures in h1 and h2, these structures have very low average connectivities, which results in low shear stiffness. Structures with a high ratio of bulk modulus to shear modulus are potential candidates for pentamode metamaterials, and the structure in inset b4 is as such already identified in the main manuscript.

Table S1. Unit cells of all structures shown in the main manuscript in the perspective view, the top view, the right side view, and the front view.

Name	Axonometric view	Top view	Right side view	Front view
	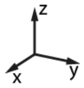	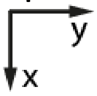	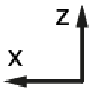	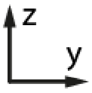
cub_Z03.0_R2234	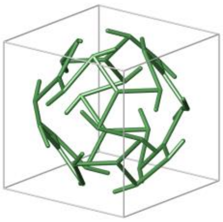	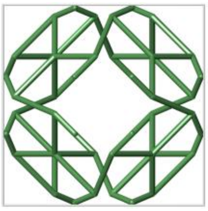	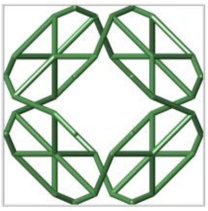	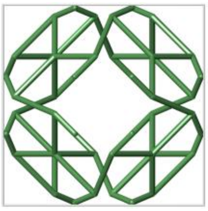
hex_Z04.3_R529	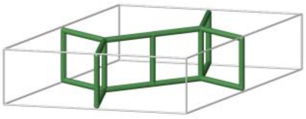	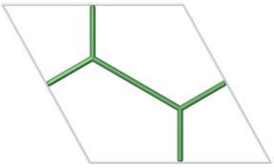	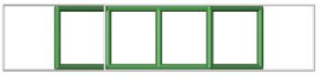	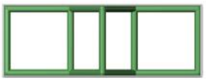
cub_Z07.2_R972	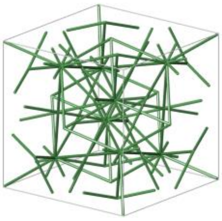	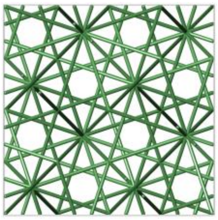	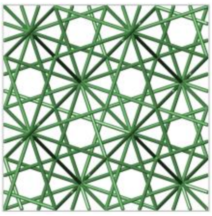	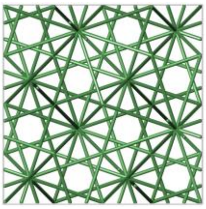
cub_Z07.4_R679	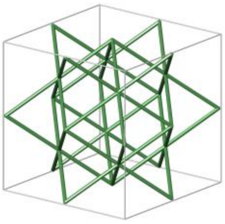	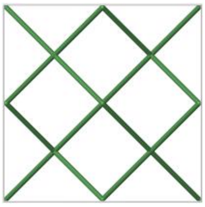	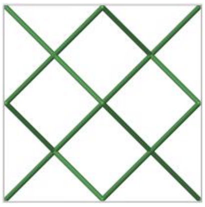	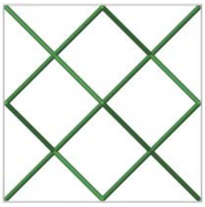
cub_Z03.6_R487	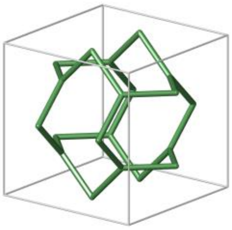	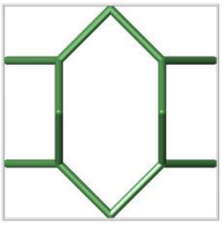	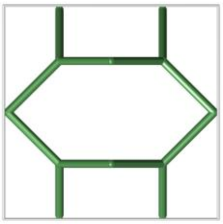	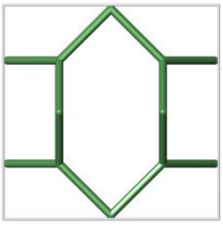
ort_Z04.0_R193	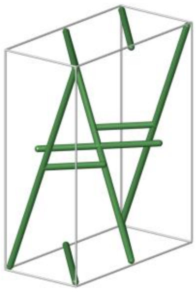	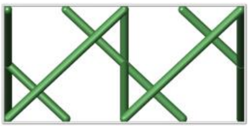	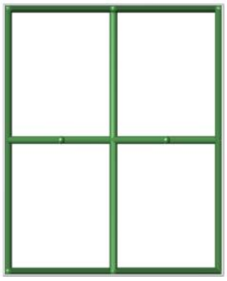	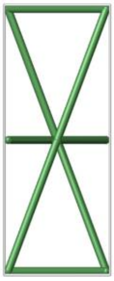

cub_Z06.0_E1<br>cub_Z06.0_R399	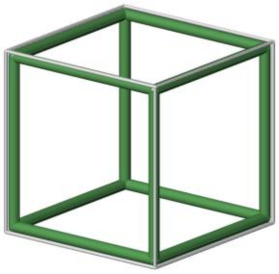	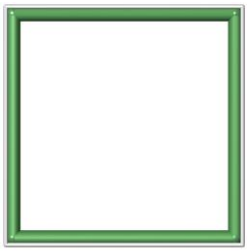	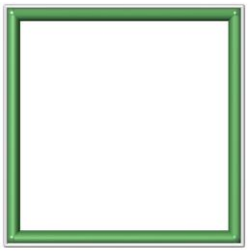	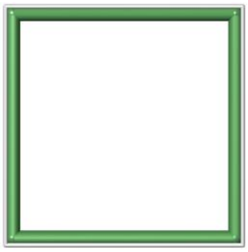
cub_Z08.0_E3<br>cub_Z08.0_R622	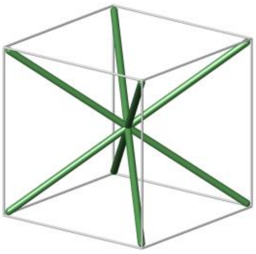	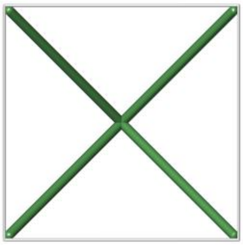	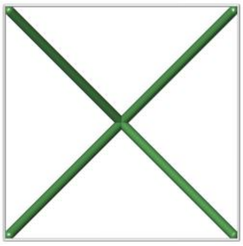	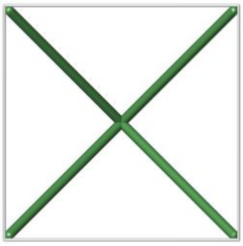
cub_Z12.0_E19<br>cub_Z12.0_R667	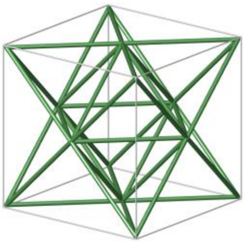	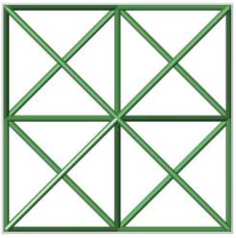	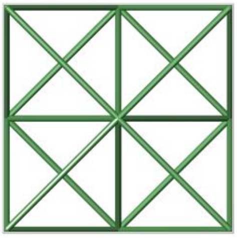	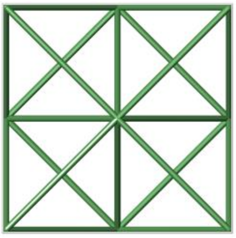
cub_Z14.0_E38<br>cub_Z14.0_R670	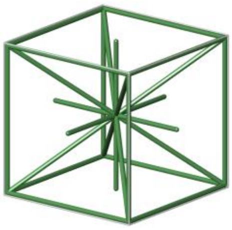	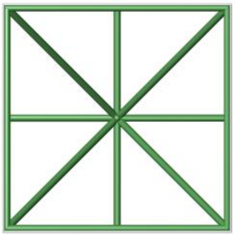	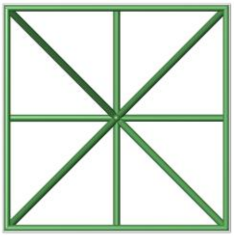	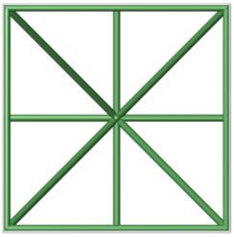
cub_Z04.0_E970<br>cub_Z04.0_R109	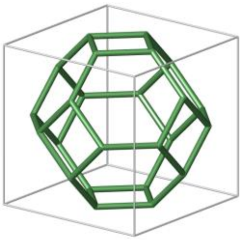	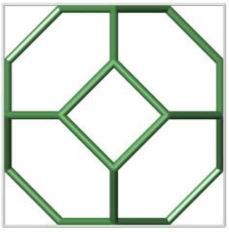	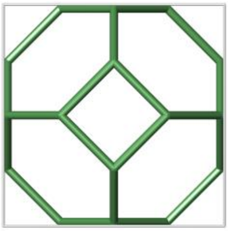	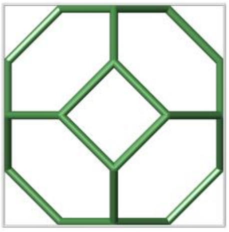
ort_Z03.8_E4453	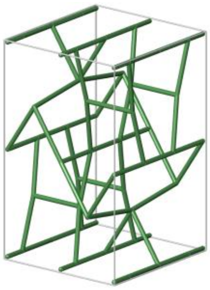	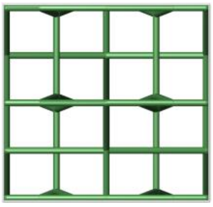	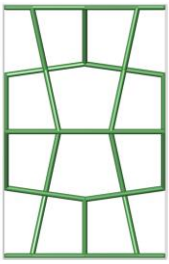	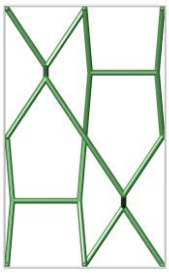

mon_Z04.3_E4603	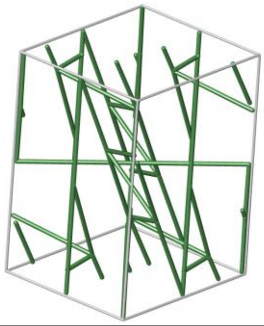	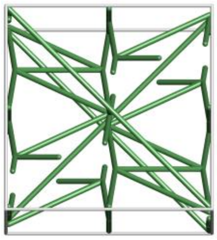	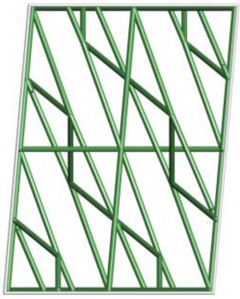	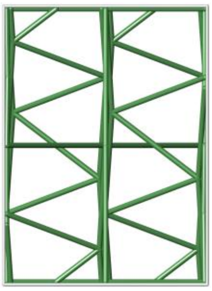
tet_Z03.8_E5841	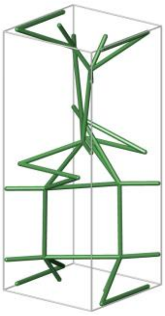	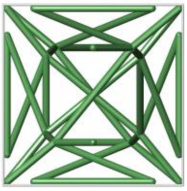	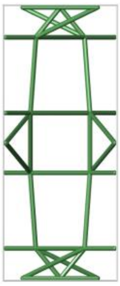	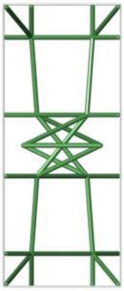
mon_Z04.0_E8038	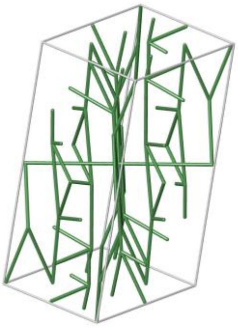	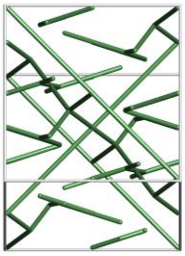	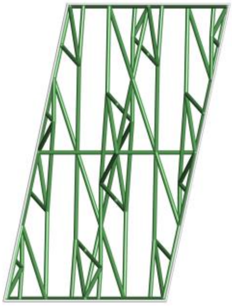	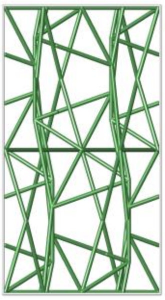
cub_Z03.6_E9066	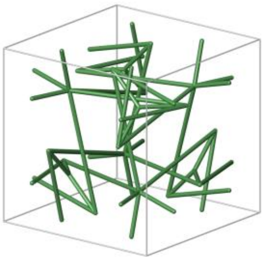	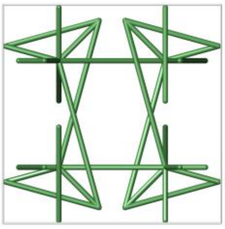	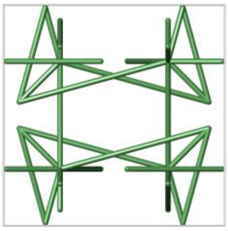	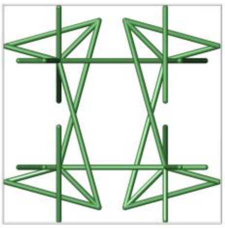
tet_Z04.3_E11423	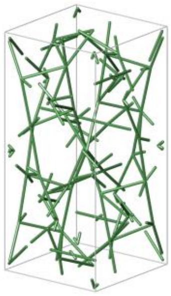	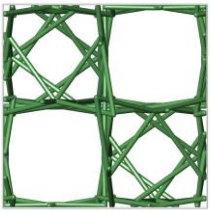	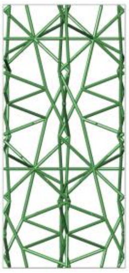	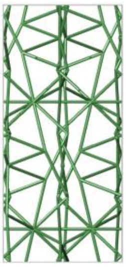
cub_Z06.0_E12702<br>cub_Z06.0_R417	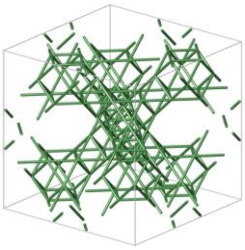	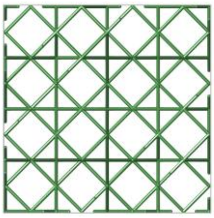	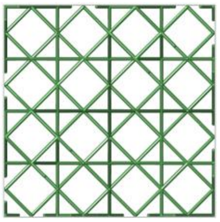	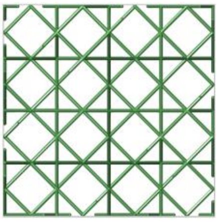

cub_Z04.1_E13435	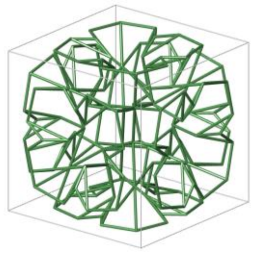	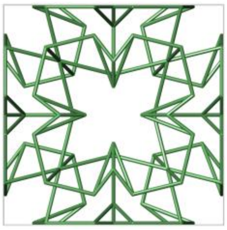	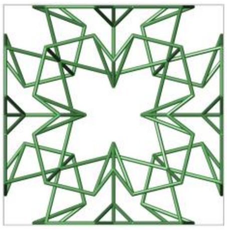	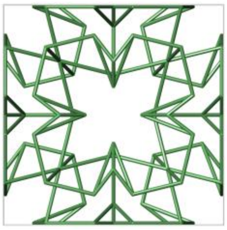
cub_Z04.0_E13519<br>cub_Z04.0_R90	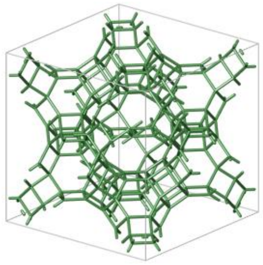	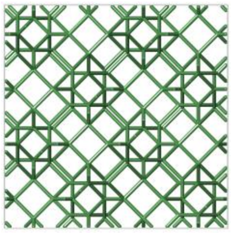	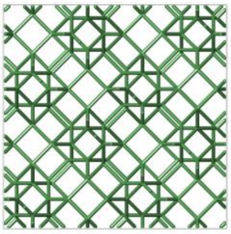	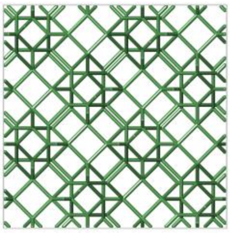
cub_Z04.5_E14462	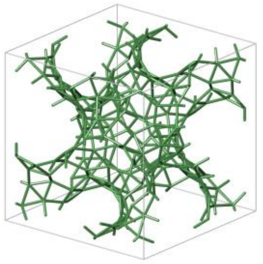	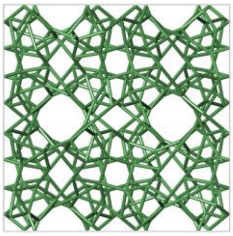	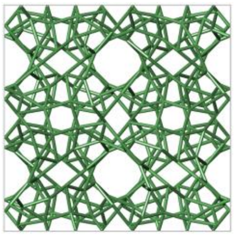	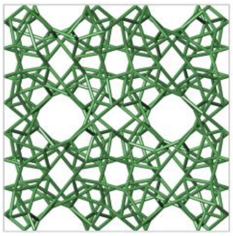

Table S2. Effective material properties of all structures shown in the paper.

Name	E_x (MPa)	E_y (MPa)	E_z (MPa)	G_{yz} (MPa)	G_{xz} (MPa)	G_{xy} (MPa)	ν_{yz} (-)	ν_{xz} (-)	ν_{xy} (-)	ν_{zy} (-)	ν_{zx} (-)	ν_{yx} (-)
cub_Z03.0_R2234	5.96E-06	5.96E-06	5.96E-06	4.00E-05	4.00E-05	4.00E-05	0.491	0.491	0.491	0.491	0.491	0.491
hex_Z04.3_R529	2.44E-05	2.44E-05	4.54E-03	1.34E-05	1.34E-05	6.13E-06	0.000	0.000	0.991	0.000	0.000	0.991
cub_Z07.2_R972	2.09E-03	2.09E-03	2.09E-03	3.23E-04	3.23E-04	3.23E-04	0.186	0.186	0.186	0.186	0.186	0.186
cub_Z07.4_R679	1.06E-05	1.06E-05	1.06E-05	1.11E-03	1.11E-03	1.11E-03	0.498	0.498	0.498	0.498	0.498	0.498
cub_Z03.6_R487	2.81E-05	2.81E-05	2.81E-05	9.86E-06	9.86E-06	9.86E-06	0.496	0.496	0.496	0.496	0.496	0.496
ort_Z04.0_R193	3.24E-06	2.41E-06	1.21E-04	5.29E-04	1.89E-06	1.26E-03	0.139	-0.161	1.160	6.985	-6.010	0.860
cub_Z06.0_E1<br>cub_Z06.0_R399	3.34E-03	3.34E-03	3.34E-03	5.31E-06	5.31E-06	5.31E-06	0.000	0.000	0.000	0.000	0.000	0.000
cub_Z08.0_E3<br>cub_Z08.0_R622	9.18E-06	9.18E-06	9.18E-06	1.11E-03	1.11E-03	1.11E-03	0.499	0.499	0.499	0.499	0.499	0.499
cub_Z12.0_E19<br>cub_Z12.0_R667	1.11E-03	1.11E-03	1.11E-03	8.34E-04	8.34E-04	8.34E-04	0.333	0.333	0.333	0.333	0.333	0.333
cub_Z14.0_E38<br>cub_Z14.0_R670	1.88E-03	1.88E-03	1.88E-03	5.96E-04	5.96E-04	5.96E-04	0.217	0.217	0.217	0.217	0.217	0.217
cub_Z04.0_E970<br>cub_Z04.0_R109	5.95E-05	5.95E-05	5.95E-05	1.96E-05	1.96E-05	1.96E-05	0.491	0.491	0.491	0.491	0.491	0.491
ort_Z03.8_E4453	2.76E-03	9.81E-06	1.65E-03	1.25E-05	8.53E-06	2.23E-06	0.054	-0.266	6.749	9.089	-0.159	0.024
mon_Z04.3_E4603	1.99E-06	2.09E-06	1.20E-03	9.44E-05	2.83E-05	2.96E-04	-0.033	0.033	0.971	-19.129	19.697	1.023
tet_Z03.8_E5841	4.65E-06	4.65E-06	2.29E-04	2.02E-06	2.02E-06	2.99E-06	-0.068	-0.068	0.354	-3.332	-3.332	0.354
mon_Z04.0_E8038	1.48E-06	1.46E-06	1.98E-03	8.42E-06	7.19E-06	2.15E-04	0.016	-0.016	1.004	21.758	-21.425	0.989
cub_Z03.6_E9066	1.10E-05	1.10E-05	1.10E-05	8.59E-07	8.59E-07	8.59E-07	0.493	0.493	0.493	0.493	0.493	0.493
tet_Z04.3_E11423	4.07E-04	4.07E-04	7.81E-04	4.12E-06	4.12E-06	7.80E-07	0.066	0.066	0.016	0.126	0.126	0.016
cub_Z06.0_E12702<br>cub_Z06.0_R417	7.43E-04	7.43E-04	7.43E-04	5.58E-04	5.58E-04	5.58E-04	0.334	0.334	0.334	0.334	0.334	0.334
cub_Z04.1_E13435	8.43E-05	8.43E-05	8.43E-05	1.70E-06	1.70E-06	1.70E-06	-0.257	-0.257	-0.257	-0.257	-0.257	-0.257
cub_Z04.0_E13519<br>cub_Z04.0_R90	5.22E-05	5.22E-05	5.22E-05	3.03E-05	3.03E-05	3.03E-05	0.491	0.491	0.491	0.491	0.491	0.491
cub_Z04.5_E14462	8.01E-05	8.01E-05	8.01E-05	2.40E-05	2.40E-05	2.40E-05	0.460	0.460	0.460	0.460	0.460	0.460

Video S1 (separate file). 3D unit cells of all structures in the Tables S1 and S2.

SI References

1. M. O'Keeffe, M. A. Peskov, S. J. Ramsden, O. M. Yaghi, The Reticular Chemistry Structure Resource, http://rcsr.anu.edu.au, (January 30, 2020).

2. M. O'Keeffe, M. A. Peskov, S. J. Ramsden, O. M. Yaghi, The reticular chemistry structure resource (RCSR) database of, and symbols for, crystal nets. *Acc. Chem. Res.* **41**, 1782–1789 (2008).

3. S. J. Ramsden, V. Robins, S. T. Hyde, S. Hungerford, EPINET: Euclidean Patterns in Non-Euclidean Tilings, http://epinet.anu.edu.au, (January 30, 2020).

4. S. J. Ramsden, V. Robins, S. T. Hyde, Three-dimensional Euclidean nets from two-dimensional hyperbolic tilings: Kaleidoscopic examples. *Acta Crystallogr. Sect. A Found. Crystallogr.* **65**, 81–108 (2009).

5. O. Delgado-Friedrichs, Program SYSTRE 19.6.0, http://rcsr.net/systre, (January 30, 2020).

6. O. Delgado-Friedrichs, Barycentric Drawings of Periodic Graphs in *Graph Drawing*, G. Liotta, Ed. (Springer Berlin Heidelberg, 2003), pp. 178–189.

7. T. S. Lumpe, T. Stankovic, Exploring the Property Space of Periodic Cellular Structures Based on Crystal Networks, Unit Cell Catalog (2020) https://doi.org/10.3929/ethz-b-000457598.

8. T. S. Lumpe, T. Stankovic, Exploring the Property Space of Periodic Cellular Structures Based on Crystal Networks, Visualization of the Unit Cell Catalog (2020) https://doi.org/10.3929/ethz-b-000457595.

9. L. A. Shuvalov, *Modern crystallography IV: physical properties of crystals* (Springer Berlin Heidelberg, 1988).

10. G. D. Cheng, Y. W. Cai, L. Xu, Novel implementation of homogenization method to predict effective properties of periodic materials. *Acta Mech. Sin.* **29**, 550–556 (2013).

11. Q. S. Yang, W. Becker, Numerical investigation for stress, strain and energy homogenization of orthotropic composite with periodic microstructure and non-symmetric inclusions. *Comput. Mater. Sci.* **31**, 169–180 (2004).

12. A. F. Bower, *Applied mechanics of solids* (CRC press, 2009).

13. C. M. Portela, J. R. Greer, D. M. Kochmann, Impact of node geometry on the effective stiffness of non-slender three-dimensional truss lattice architectures. *Extrem. Mech. Lett.* **22**, 138–148 (2018).

14. V. S. Deshpande, N. A. Fleck, M. F. Ashby, Effective properties of the octet-truss lattice material. *J. Mech. Phys. Solids* **49**, 1747–1769 (2001).

15. R. M. Sullivan, L. J. Ghosn, B. A. Lerch, A general tetrakaidecahedron model for open-celled foams. *Int. J. Solids Struct.* **45**, 1754–1765 (2008).

16. M. A. Meyers, K. K. Chawla, *Mechanical Behavior of Materials* (Cambridge University Press, 2009).

17. J. F. Nye, *Physical properties of crystals: their representation by tensors and matrices* (Oxford University Press, 1985).

18. M. O'Keeffe, B. G. Hyde, *Crystal Structures. I. Patterns and Symmetry* (Mineralogical Society of America, Washington, D.C., 1996).

19. L. R. Meza, *et al.*, Reexamining the mechanical property space of three-dimensional lattice

architectures. *Acta Mater.* **140**, 424–432 (2017).

20. V. S. Deshpande, M. F. Ashby, N. A. Fleck, Foam Topology Bending Versus Stretching Dominated Architectures. *Acta Mater.* **49**, 1035–1040 (2001).

21. Z. Hashin, S. Shtrikman, A variational approach to the theory of the elastic behaviour of multiphase materials. *J. Mech. Phys. Solids* **11**, 127–140 (1963).

22. E. Andreassen, B. S. Lazarov, O. Sigmund, Design of manufacturable 3D extremal elastic microstructure. *Mech. Mater.* **69**, 1–10 (2014).
